# Metalloproteinase-9 contributes to endothelial dysfunction in atherosclerosis via protease activated receptor-1

**DOI:** 10.1371/journal.pone.0171427

**Published:** 2017-02-06

**Authors:** Jon M. Florence, Agnieszka Krupa, Laela M. Booshehri, Timothy C. Allen, Anna K. Kurdowska

**Affiliations:** 1 Department of Cellular and Molecular Biology, University of Texas Health Science Center at Tyler, Tyler, Texas, United States of America; 2 Institute of Medical Biology, Polish Academy of Sciences, Lodz, Poland; 3 Department of Pathology, University of Texas Medical Branch, Galveston, Texas, United States of America; Universita degli Studi Magna Graecia di Catanzaro Scuola di Medicina e Chirurgia, ITALY

## Abstract

The atherosclerotic process begins when vascular endothelial cells undergo pro-inflammatory changes such as aberrant activation to dysfunctional phenotypes and apoptosis, leading to loss of vascular integrity. Our laboratory has demonstrated that exposure of mice to second hand smoke triggers an increase in expression of metalloproteinase-9. Further, metalloproteinase-9 released by second hand smoke—activated leukocytes may propagate pro-atherogenic alterations in endothelial cells. We have shown that levels of metalloproteinase-9 were increased in the plasma from apolipoprotein E deficient (ApoE^-/-^) mice exposed to second hand smoke relative to non-exposed controls. Moreover, we have collected data from two different, but complementary, treatments of second hand smoke exposed atherosclerotic mice. Animals received either cell specific metalloproteinase-9 directed siRNA to minimize metalloproteinase-9 expression in neutrophils and endothelial cells, or a pharmacological inhibitor of Bruton’s tyrosine kinase which indirectly limits metalloproteinase-9 production in neutrophils. These treatments reduced atherosclerotic changes in mice and improved overall vascular health. We also demonstrated that metalloproteinase-9 could activate endothelial cells and induce their apoptosis via cleavage of protease activated receptor-1. In summary, better understanding of metalloproteinase-9’s pathogenic capabilities as well as novel signaling pathways involved may lead to development of treatments which may provide additional benefits to atherosclerosis patients with a history of second hand smoke exposure.

## Introduction

Atherosclerosis, a chronic, inflammatory, fibroproliferative disease affecting large- and medium- sized arteries, is the common denominator in most cardiovascular diseases, including coronary artery disease, heart failure, and stroke—major causes of death in industrialized countries [1–3]. Potential causes of atherosclerosis include smoking, hypertension, hypercholesterolemia, diabetes, obesity, chronic infections, and chronic low-grade inflammation. Atherosclerosis begins with pro-inflammatory endothelial cell changes, leading to endothelial cell activation; the first manifestation of endothelial cell dysfunction [1–3]. Most mediators of cardiovascular disease pathogenesis activate endothelial cells, causing a substantial increase in expression of chemokines, cytokines, adhesion molecules, and other pro-inflammatory agents. Leukocyte infiltration, an essential step in atherosclerotic lesion formation, then occurs, with consequent loss of vascular integrity. Apoptosis causes endothelial cell damage and cellular detachment; therefore, atherosclerotic patients have increased levels of circulating endothelial cells and endothelial microparticles [4–6].

Matrix metalloproteinases (MMPs) are a group of proteolytic enzymes capable of degrading extracellular matrix components such as elastin, proteoglycans, and collagen. Activity of MMPs is regulated by proteinase inhibitors, i.e., tissue inhibitors of metalloproteinases (TIMPs) and α-2-macroglobulin. All of the 30 known mammalian MMPs participate in various biological phenomena associated with vascular remodeling. MMPs have the ability to alter blood vessel size and composition; processes essential for vessel plasticity and repair. However, excessive MMP release may lead to inappropriate cardiovascular remodeling and atherosclerosis. MMPs have been detected in atherosclerotic plaques, and in close proximity to foam cell clusters—clusters of macrophages that engulfed lipoprotein particles [7–9].

In particular, metalloproteinase-9 (MMP-9, gelatinase B, or type IV collagenase), has been implicated in the pathogenesis of atherosclerosis [7]. Nucleotide polymorphisms of the MMP-9 gene are associated with the presence and severity of atherosclerosis; and increased circulating levels of MMP-9 have been identified in type 2 diabetes patients with coronary artery disease. In addition, increased circulating levels of MMP-9 correlate with cardiovascular risk factors, and are linked to development of the stroke and other serious cardiovascular events such as acute myocardial infarction and unstable angina. Further, the MMP-9 concentration in plasma coronary artery disease patients predicts cardiovascular mortality. MMP-9 is involved in all stages of atherosclerosis. MMP-9 may also promote transmigration of monocytes across the endothelium by disrupting the basement membrane surrounding endothelial cells. In addition, *in vitro* studies show that both vascular injury and endothelial cell-monocyte interaction may trigger an increase in MMP-9 expression. Macrophage foam cells and smooth muscle cells (SMCs) also secrete MMP-9 in response to inflammatory mediators. Moreover, MMP-9 is capable of degrading the extracellular matrix around SMCs facilitating SMC migration into the atherosclerotic lesion where they proliferate and secrete collagen along with other extracellular matrix proteins required for the formation of the fibrous cap. Finally, MMP-9 may contribute to destabilization of the plaque [7–9].

Hypothesizing that MMP-9 directly affects endothelial cells, we use a mouse model to precisely define the pro-inflammatory and pro-apoptotic activity of second hand smoke (SHS) in a reliable, well-characterized *in vivo* system.

## Methods

### Animal studies

All studies involving animals were approved by the IACUC at the University of Texas Health Science Center, and conform to National Institutes of Health guidelines. The IACUC protocol included provisions for the early euthanasia of animals who became severely ill / moribund during the experiment (signs of illness or distress e.g. labored breathing, huddling, neglect of grooming). Condition of animals was monitored by laboratory staff before and after SHS exposure and treatment, 4 to 6 times per day respectively, and also by vivarium staff at least once daily. No animals appeared to be ill throughout the experiments. Animals were euthanized by intraperitoneal injection of Beuthanasia (pentobarbital sodium, phenytoin sodium combination solution). In addition, three animals total died before treatments were concluded (two from group treated with siRNA specific for MMP-9 (Invitrogen) conjugated with F(ab)_2_ fragments of anti-mouse neutrophil antibody (clone Ly-6G1A8) and one from group treated with siRNA specific for MMP-9 conjugated with anti-mouse endothelial cell antibody [clone MECA-32]). Cause of death was not determined.

Age matched female MPF (Murine Pathogen Free) apolipoprotein E deficient (ApoE^-/-^, C57Bl/6) mice and RF (Restricted Flora) C57BL/6 wild type mice (Taconic; Germantown, NY) were used in this study. ApoE^-/-^ mice were divided into four treatment groups consisting of (1) animals fed a high fat, high cholesterol Western Diet (WD) (D12079B, Research Diets Inc.; New Brunswick, NJ) with SHS exposure; (2) WD fed animals without SHS exposure; (3) standard rodent chow (Chow) (PicoLab Rodent Diet 20, LabDiet; St. Louis, MO) fed animals with SHS exposure; and (4) Chow fed animals without SHS exposure (4). Wild type C57Bl/6 mice were divided into two treatment groups, (1) Chow fed animals with SHS exposure; and (2) Chow fed animals without SHS exposure. Mice were exposed to passive cigarette smoke using a whole body smoke exposure system (TE-10B, Teague Enterprises; Woodland, CA), as previously described [10]. Briefly, mice were exposed to a combination of 11% mainstream and 89% side stream smoke from 40 3R4F reference cigarettes twice daily, 5 days a week, for indicated times. Control animals were exposed to room air only. Treatment animals were injected intravenously (i.v.) [tail vein injection] with either (1) Bruton’s tyrosine kinase inhibitor (BTK Inh.) PCI-32765 (Selleck Chemicals) or (2) siRNA specific for MMP-9 (Invitrogen) conjugated with F(ab)_2_ fragments of anti-mouse neutrophil antibody (clone Ly-6G1A8), or anti-mouse endothelial cell antibody (clone MECA-32) in PBS. Treatments were started after 7 weeks of WD+SHS exposure, and animals were treated twice per week for 2 or 4 weeks while continuing regular WD+SHS exposure. Control animals were exposed only to WD+SHS throughout the experiments. Five to six animals per treatment group, and four to five animals per control group were used.

Following periods of SHS exposure, mice were euthanized and the thoracic cavity was opened; blood was collected directly from the right ventricle using sodium citrate as an anticoagulant. Plasma was separated from cells and platelets by centrifugation, aliquoted, frozen under dry ice, and stored at -70°C. The renal vein was then cut and excess blood was flushed from the vasculature through the heart using 20 ml cold PBS. Under a dissecting microscope the intact aortic arch was isolated as previously described [11], frozen in Tissue-Tek OCT compound (Sakura Finetek; Torrance, CA), and sectioned at 5 μM. These mice were also used for experiments, involved with COPD, in a recently submitted study [Novel Treatments for Severe Chronic Lung Inflammation. Respiratory Research].

### Histochemical evaluation of atherosclerotic lesions

For histochemical staining, aortic arch sections were fixed in PBS containing 4% paraformaldehyde and then stained with Oil Red O (ORO), Picro-Sirius red (PS), and by Russel-Movat’s Pentachrome method. All stained sections were then digitally photographed. For plaque/lesion area analysis Oil Red O (ORO) stained sections were quantified by selecting the ORO-positive areas within a 2.2-mm segment of lesser curvature and innominate artery lesions and measuring the area using ImageJ software. PS red sections were likewise quantified with ImageJ by first selecting the lesions in indicated regions of the aortic arch and measuring the total area, then applying a bright-scale threshold to the same region to select only areas with positive staining visible under polarized light and measuring the corresponding area. Percent collagen content was estimated by dividing PS red positive area by total area. Movat’s pentachrome stained sections were quantified by histological scoring of tissue sections. For collagen density scoring each lesion in the indicated region was given a score ranging from 1–4. Four indicated that collagen positive areas were solid bodies with little to no collagen strands surrounding empty space, 3 indicated a mostly solid lesion with some thin strands surrounding empty space, 2 indicated layers of thin strands with some solid regions of collagen present, and 1 indicated only thin strands of collagen visible. If no plaque was present for a given region/section no score was assigned. Matrix positive stain (blue-green color) scoring was done in a similar manner. A score of 4 indicated positive matrix staining throughout the lesion, 3 indicated multiple regions within the lesion with matrix positive staining, 2 indicated some matrix positive regions in a majority matrix negative lesion, and 1 indicated practically no positive staining for a given lesion. If no plaque was present for a given region/section no score was assigned.

### ELISA assays

Plasma was assayed for concentrations of KC, monocyte chemotactic protein-1 (MCP-1) and adhesion molecules (intercellular adhesion molecule, ICAM-1) [R&D Systems; Minneapolis, MN] according to manufacturer instructions. In addition, plasma samples were diluted 50x in assay diluent (1% BSA/PBS) and assayed in duplicate using the R&D systems DuoSet mouse CRP (C-reactive protein), ELISA kit according to manufacturer instructions.

### MMP-9 assays: Gelatin zymography and enzyme activity assay

MMP-9 enzymatic activity was assayed in plasma by both traditional gelatin zymography and a mouse MMP-9 specific enzymatic activity assay (QuickZyme Biosciences; Leiden, The Netherlands) according to manufacturer instructions. For zymography, equal volumes of plasma from 4–6 mice per group were pooled together and denatured in non-reducing 6x Laemmli sample buffer at a ratio of 1:5. Equal volumes equivalent to 3 μl of undiluted plasma samples were then separated on 8% polyacrylamide gels containing 0.05% gelatin under constant voltage. After electrophoresis, gels were removed from cassettes and enzymes were allowed to re-fold in Novex Zymogram Renaturing Buffer (Life Technologies; Grand Island, NY) at room temperature for 90 minutes, replacing buffer every 30 minutes. Gels were then rinsed and incubated at 37°C in Novex Zymogram Developing Buffer (Life Technologies; Thermo Fisher Scientific; Waltham MA) containing 0.02% sodium azide for 90 hours. Following incubation, gels were soaked for 45 minutes in 20% trichloroacetic acid, then stained with Brilliant Blue R250 (Sigma Aldrich; St. Louis, MO) and de-stained until zones of digestion were visible as clear bands against a blue background.

### HUVEC cell culture conditions

Human Umbilical Vein Endothelial Cells (HUVEC) (Cambrex; Walkersville, MD) were cultured in endothelial basal medium enriched with endothelial growth factor, hydrocortisone, bovine brain extract, heparin, fetal bovine serum, antibiotics (gentamicin and amphotericin B), and endothelial cell growth media (EGM-MV, Cambrex; East Rutherford, NJ) as performed routinely in our laboratory [12]. Cells were used for experiments between passages 2 and 15. They were maintained in plastic culture flasks or tissue culture Petri dishes in a humidified atmosphere of 5% CO_2_ at 37°C.

### Western blotting

HUVEC cells were incubated for 5 minutes with human enzymatically active MMP-9 at the concentration range of 0.1–0.01 nM (Calbiochem; San Diego CA). Cells were stimulated in medium without serum containing sodium orthovanadate (1 mM) (Sigma Aldrich; St. Louis, MO) at 37°C in 5% CO_2_. After 5 minutes, cells were lysed with 4x Laemmli Sample Buffer prepared in the laboratory according to the Bio-Rad (Hercules, CA) protocol containing 0.5% β-Mercaptoethanol (Thermo Fisher Scientific; Waltham MA), then boiled for 3 minutes before storing at -70°C. Samples were subsequently loaded onto a SDS-PAGE gel, and separated proteins were transferred to a polyvinylidene difluoride membrane (Pall Corp.; Pensacola, FL). The membrane was blocked and incubated with anti-pERK (Thr202/Tyr204) antibody and anti-total ERK (L34F12) (Cell Signaling Technology; Danvers, MA), and anti-Actin antibody (I-19, Santa Cruz Biotechnology; Dallas, TX) followed by the appropriate HRP conjugated secondary antibody. Bound antibodies were detected using enhanced chemiluminescence reagents (PerkinElmer Life Sciences, Inc.; Boston, MA). The membrane was then exposed to X-ray film (FGX810; Phenix; Hayward, CA).

### In vitro MMP-9 induced HUVEC apoptosis

Apoptosis was examined through observed caspase 3 enzymatic activity in lysates of HUVEC cells cultured with and without MMP-9 using the fluorescent substrate Ac-DEVD-AFC (Sigma Aldrich; St. Louis, MO) based on manufacturer instructions.

### Cell signaling visualized by confocal microscopy

HUVEC cells cultured on coverslips were incubated for 5 minutes with human MMP-9 (Calbiochem; San Diego, CA) at the concentration range of 0.1–0.01 nM in medium without serum containing sodium orthovanadate (1 mM) (Sigma Aldrich; St. Louis, MO). In some experiments HUVEC cells were treated with monoclonal antibodies against PAR1 receptor (WEDE15 and ATAP2; Immunotech Laboratories Inc.; Monrovia, CA and Santa Cruz Biotechnology; Dallas, TX, respectively) for 30 minutes at 4°C prior to incubation with human MMP-9. After 5 minutes of stimulation with human MMP-9, the cells were washed with HBSS without Ca^2+^ and Mg^2+^, fixed with 100% methanol, permeabilized with 0.5% (vol/vol) Triton X-100 in PBS, blocked in 5% BSA in PBS, and incubated overnight at 4°C with anti-pERK (Thr202/Tyr204) antibody (Cell Signaling Technology; Danvers, MA). To visualize phosphorylation of ERK, HUVEC cells were then incubated with Alexa Fluor 488 conjugated chicken anti-rabbit antibody (Invitrogen; Waltham, MA). The cells were observed using Perkin Elmer Ultra VIEW LCI confocal imaging system with Nikon TE2000-S fluorescence microscope and PlanApo360 immersion oil objective (numerical aperture [NA] 1.4) at room temperature. Ultra VIEW Imaging Suite software (version 5.5.0.4) was used for image processing.

### Protease activated receptor-1 cleavage

HUVEC cells cultured on coverslips were stimulated with human MMP-9 for 5 minutes, as described above. The cells were then washed with HBSS without Ca^2+^ and Mg^2+^, fixed with 100% methanol, blocked with 5% BSA in PBS followed by incubation with anti-PAR-1 receptor antibody (ATAP-2, Zymed Laboratories Inc.; Bedminster, NJ). Anti-PAR-1 (ATAP-2) antibody recognizes intact and cleaved forms of PAR-1 receptor. Finally, cells were incubated with Alexa Fluor 647 conjugated goat anti-mouse secondary antibody (Invitrogen; Waltham, MA). Cleavage of PAR-1 receptor was visualized using Perkin Elmer Ultra VIEW LCI confocal imaging system with Nikon TE2000-S fluorescence microscope and PlanApo360 immersion oil objective (numerical aperture 1.4) at room temperature. Ultra VIEW Imaging Suite software (version 5.5.0.4) was used for image processing.

### Analysis of human aortic tissue sections

Aortic leaflets were collected for pathological evaluation by the Department of Pathology, University of Texas Health Science Center at Tyler, and all patient specimens were de-identified and archived. We obtained an Exempt Protocol from the Institutional Review Board (IRB), University of Texas Health Science Center, Tyler, TX. Written consent for research use of tissue obtained during hospitalization was obtained at the time of hospital admission as part of the patient's general consent for treatment. Consent was therefore documented via the patient's general consent for hospital admission and treatment. Verbal consent was not obtained. This method of obtaining consent was approved by the IRB.

Tissues were fixed and embedded in paraffin. For analysis, 15 μm sections were placed on silanized slides, dried in an oven at 60°C, and deparaffinized by running the tissue section slides through multiple changes of xylene, and then 100%, 95%, and 70% alcohols down to distilled water. All of aortic leaflets and normal aortic tissues specimens were evaluated for the expression of cleaved caspase 3 (rabbit anti-human cleaved caspase 3, Cell Signaling), PAR-1 (goat anti-human PAR-1, Santa Cruz Biotechnology), and active MMP-9 (mouse anti-human active MMP-9, Santa Cruz Biotechnology). CD34 (anti-human CD34, Santa Cruz Biotechnology) was used as an endothelial cell marker. After incubation with appropriate secondary antibodies (Invitrogen), human tissues were evaluated using a PerkinElmer Ultra VIEW LCI confocal imaging system with Nikon TE2000-S fluorescence microscope equipped with PlanApo320 objective, and PlanApo360, or 3100 immersion oil objective (NA, 1.4) at room temperature. Ultra VIEW Imaging Suite software (version 5.5.0.4) was used for image processing.

### Statistics

Differences between groups were evaluated by a simple one-way ANOVA, or *t* test when appropriate. A *P* value of less than 0.05 was considered significant. All statistics were performed using SigmaStat (SPSS Science; Chicago, IL).

## Results

We have used a mouse model to precisely define the pro-inflammatory and pro-apoptotic activity of SHS in a reliable, well-characterized *in vivo* system. ApoE^-/-^ mice, in which the targeted deletion of the *apoE* gene leads to severe hypercholesterolemia and spontaneous atherosclerosis, are among the most widely used mouse models for atherosclerosis. Atherosclerotic lesions in ApoE^-/-^ mice resemble their human counterparts, progressing over time from initial fatty streaks to complex lesions. A high-fat, high-cholesterol diet strongly accelerates the process [13, 14]. SHS exposure is another important risk factor for atherogenesis. It is well established that SHS promotes atherosclerosis development and progression through its adverse effects on the endothelium [15].

**Fig 1:**
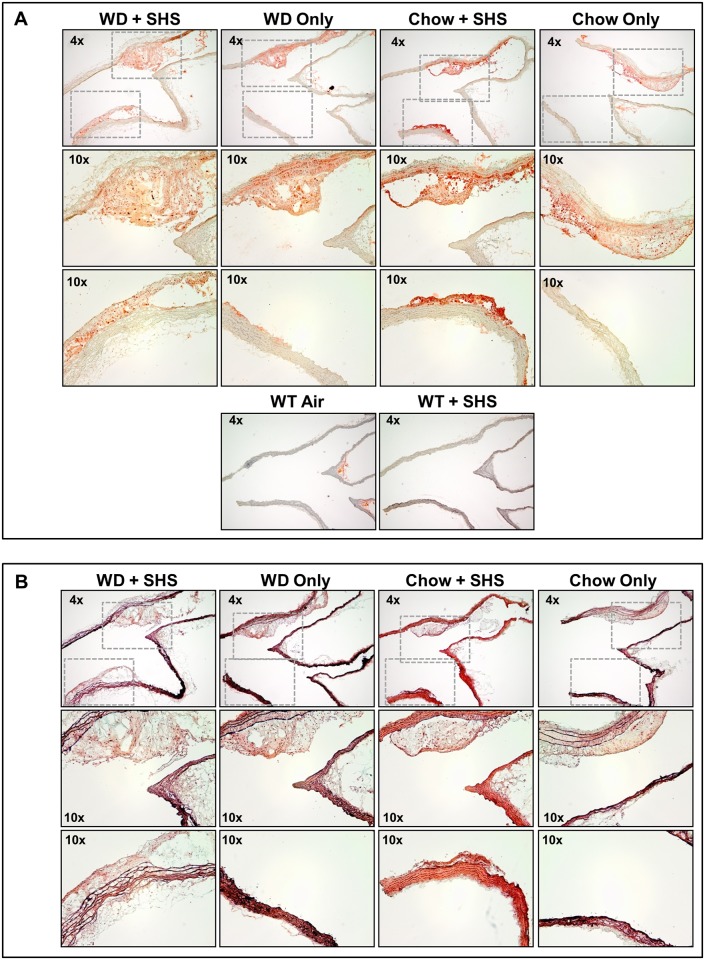
Histological analysis of aortic arch cross sections from ApoE^-/-^ mice after 7 weeks of exposure. (A) Oil Red O stained sections of aortic arch from ApoE^-/-^ mice after 7 weeks of WD and SHS exposure (WD + SHS), WD only, Chow and SHS exposure (Chow + SHS), and Chow only. Wild type mice fed Chow with and without SHS exposure are shown as controls (WT + SHS, WT Air). Three to five mice per group were examined. (B) Movat’s pentachrome stained cross sections of aortic arch from ApoE^-/-^ mice after 7 weeks of WD + SHS, WD only, Chow + SHS, and Chow only. Three to four mice per group were examined.

**Fig 2:**
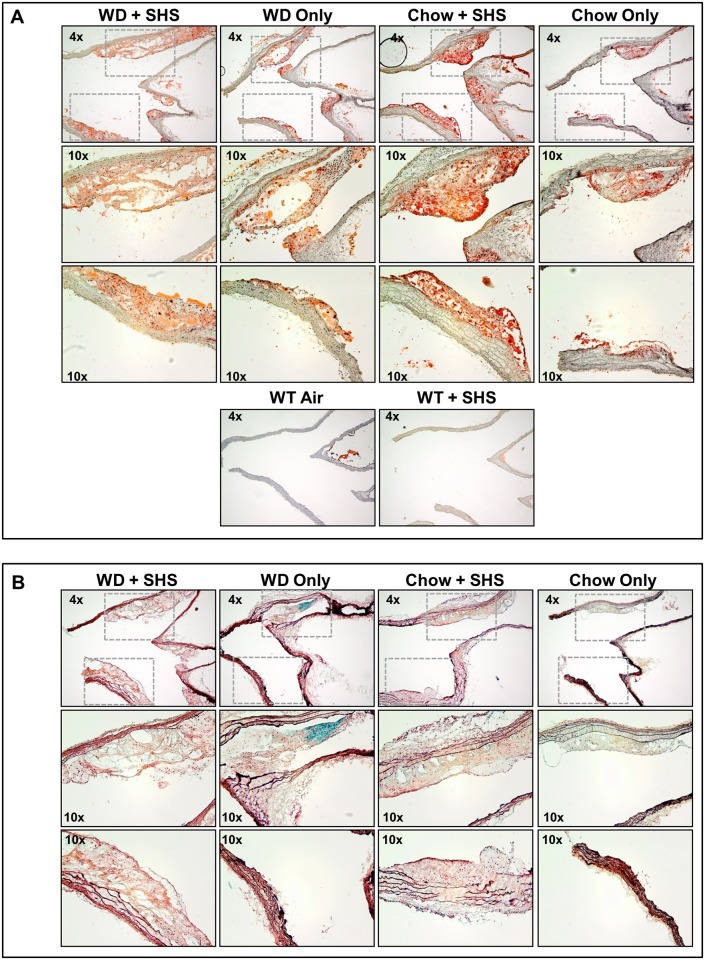
Histological analysis of aortic arch cross sections from ApoE^-/-^ mice after 13 weeks of exposure. (A) Oil Red O stained cross sections of aortic arch from ApoE^-/-^ mice after 13 weeks of WD + SHS, WD only, Chow + SHS, and Chow only. Wild type mice fed Chow with and without SHS exposure are shown as controls (WT + SHS, WT Air). Four to six ApoE^-/-^ mice per group were examined along with 3 WT mice per group. (B) Movat’s Pentachrome stained cross sections of aortic arch from ApoE^-/-^ mice after 13 weeks of WD + SHS, WD only, Chow + SHS, and Chow only. Three to four mice per group were examined.

**Fig 3:**
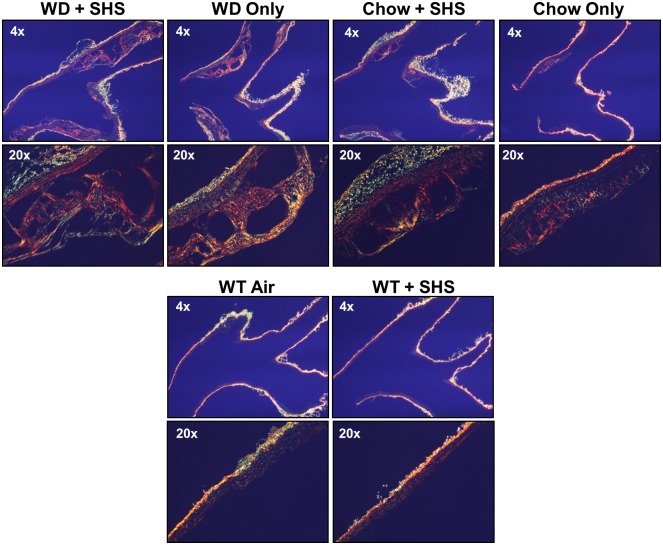
Picro-Sirius red stained cross sections of aortic arch viewed under polarized light. Aortic arch sections were examined from ApoE^-/-^ mice after 13 weeks of WD + SHS, WD only, Chow + SHS, and Chow only. Wild type mice fed rodent chow with and without smoke exposure are shown as controls (WT + SHS, WT Air). For ApoE^-/-^ mice 5–6 mice group were examined, 3 WT mice per group were examined.

Histological staining was performed on major atherosclerotic lesions contained in the aortic arch of these mice using both Oil Red O and Movat’s Pentachrome stains to confirm the presence of atherosclerotic changes (Figs 1–3). Lesion size increased in WD fed animals relative to standard chow fed controls, and lesions from SHS exposed animals appeared less compact than non-smoking controls (Figs 1A and 2A and S1 and S2 Figs; 7 and 13 weeks exposures, respectively). No lesions were identified in WT mice regardless of SHS exposure. Lipids stain red against an unstained background, and hematoxylin was used as a counterstain. In addition, collagen content and collagen density were lower in SHS exposed animals relative to non-smoking controls as indicated by reduced yellow-gold staining (Figs 1B and 2B and S2 and S4 Figs; 7 and 13 weeks exposures, respectively). We also noted a decrease in the amount of intra-lesion ground substance / extracellular matrix at 13 weeks of exposure as indicated by reduced blue-green staining (Fig 2B and S4 Fig). Picro-Sirius red collagen staining was observed under polarized light to evaluate the progression of atherosclerosis and the pathological contributions of SHS at 13 weeks of exposure (Fig 3 and S3 Fig). We confirmed that lesion size increased in WD fed animals relative to standard chow fed controls, and lesion collagen from SHS exposed animals appeared less compact than from non-smoking controls. Differences in collagen density were observed by the presence/absence of stained tissue.

**Fig 4:**
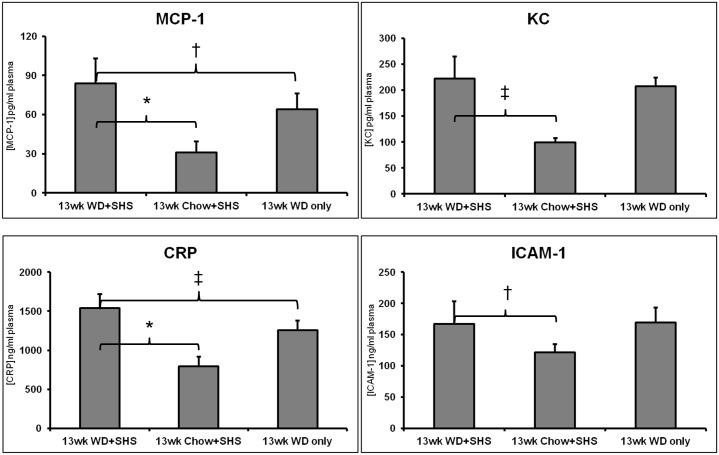
Changes in plasma levels of chemokines (KC, MCP-1, CRP and ICAM-1). Changes in plasma levels of chemokines (KC and monocyte chemotactic protein-1 [MCP-1]), C-reactive protein (CRP), and adhesion molecules (intercellular adhesion molecule, ICAM-1) at 13 weeks post WD and / or SHS exposure. Values are shown as mean ± STD, * p<0.01, ‡ p<0.05, † p<0.1. For WD + SHS group n = 5, Chow + SHS and WD Only groups n = 6.

In addition, we observed substantial changes in plasma levels of chemokines (KC and monocyte chemotactic protein-1 [MCP-1]), as well as other inflammatory mediators, including C-reactive protein (CRP) and adhesion molecules (intercellular adhesion molecule, ICAM-1) at 13 weeks post WD and/or SHS exposure, indicative of endothelial cell inflammation and activation (Fig 4).

**Fig 5:**
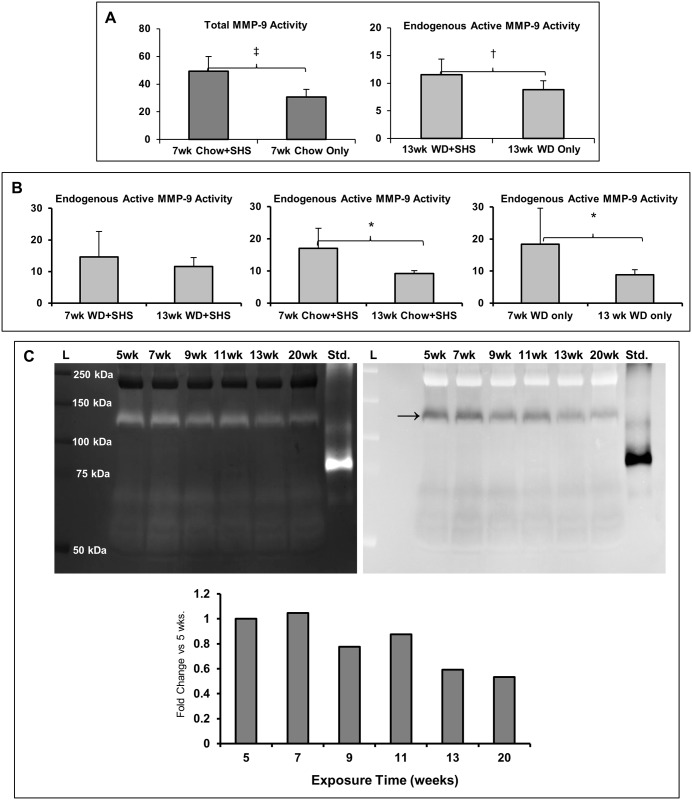
Level of MMP-9 (total and endogenous active) in plasma of WD + SHS exposed ApoE^-/-^ mice. (A) Total MMP-9 in plasma at 7 weeks and endogenous active MMP-9 at 13 weeks exposure. Values are shown as mean ± STD, † p<0.1, ‡ p<0.05. For 7 weeks groups n = 5, for 13 weeks groups n = 6. (B) Comparison of endogenous active MMP-9 in plasma after 7 and 13 weeks exposure. Values are shown as mean ± STD, * p<0.01. For 7 weeks groups n = 5, for 13 weeks groups n = 6. (C) Gelatin zymography of pooled plasma taken after 5 to 20 weeks exposure, stained gel is shown along with inversion image and corresponding densitometry analysis of the most prominent band (indicated with arrow). For pooling plasma n = 4 at 5 weeks, n = 6 at 13 weeks, and n = 5 for remaining time points.

We also measured MMP-9 concentration at 7 and 13 weeks post WD and / or SHS exposure, and identified elevated plasma concentrations of active MMP-9 in ApoE^-/-^ mice that were both fed WD and exposed to SHS at 13 weeks; whereas total MMP-9 increased in mice exposed to regular diet and SHS at 7 weeks (Fig 5A). In addition, we noted that the plasma concentrations of the active form of MMP-9 generally decreased over time (Fig 5B). Finally, gelatin zymography of plasma from WD fed, SHS exposed, ApoE^-/-^ mice clearly showed significant bands in the range of 125 kDa (Fig 5C), representing MMP-9:NGAL (neutrophil gelatinase-associated lipocalin) complexes, a form of active MMP-9 associated with neutrophil degranulation. Whether the MMP-9:NGAL complexes protect active MMP-9 from degradation by NGAL remains to be determined; however, active human MMP-9 is reportedly protected from degradation when complexed with NGAL [16–18].

Next, we employed treatments with MMP-9-directed siRNA designed to minimize cell-specific MMP-9 expression in endothelial cells and neutrophils; as well as with PCI-32765, a small molecule inhibitor of Bruton’s tyrosine kinase, which indirectly limits MMP-9 production in neutrophils [19, 20]. The animals were treated i.v. (tail vein injection) with either the Bruton’s tyrosine kinase inhibitor (BTK Inh.) PCI-32765, or siRNA specific for MMP-9 conjugated with F(ab)_2_ fragments of anti-mouse endothelial cell antibody (clone MECA-32), or anti-mouse neutrophil antibody (clone Ly-6G1A8). Treatments started at 7 weeks post WD and / or SHS exposure, as per our findings presented in Fig 5C.

**Fig 6:**
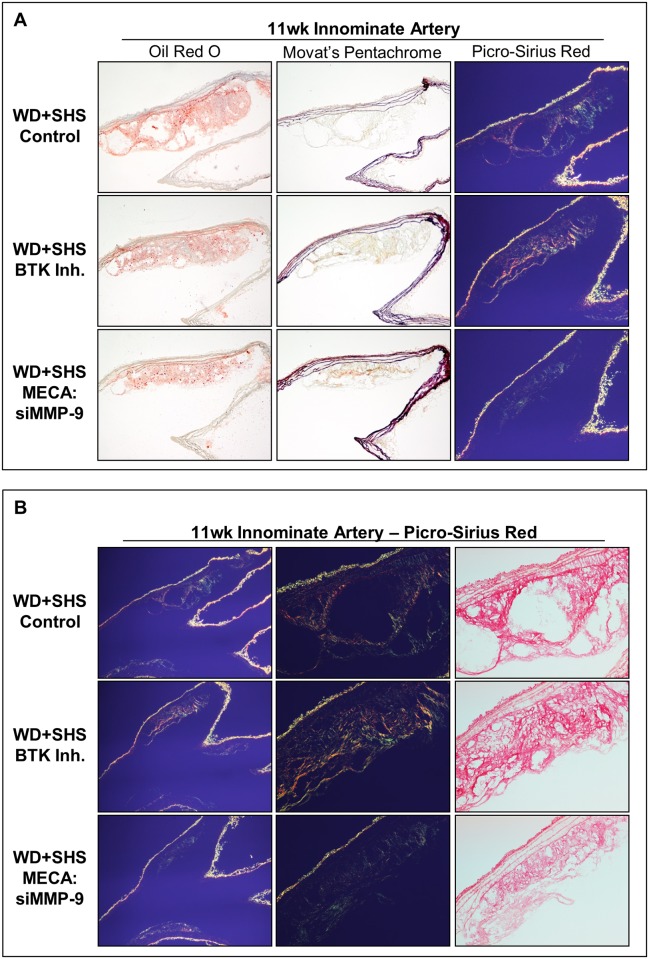
Histological analysis of aortic arch cross sections at the initial branch of the innominate artery from ApoE^-/-^ mice following four week treatments. (A) Representative Oil Red O stained sections of aortic arch show atherosclerotic lesions found at the initial branch of the innominate artery of control and treated mice; 4–8 animals per group were analyzed. Neutral lipids stained red. Movat’s Pentachrome and Picro-Sirius red stained sections of aortic arch are also shown; 3–5 mice per group were analyzed. (B) Representative Picro-Sirius red stained sections of aortic arch show atherosclerotic lesions found at the innominate artery of control and treated mice when viewed under normal (right) and polarized (left, center) light; 3–5 animals were evaluated per group.

Representative Oil Red O stained aortic arch sections show atherosclerotic lesions at the initial branch of the innominate artery of control and treated mice (Fig 6A and S5 Fig). Relative to control mice, lesion size was reduced in MECA:siMMP-9 treated animals, and to a lesser extent in BTK Inh. treated animals. Sections containing atherosclerotic lesions at the innominate artery were also stained using Movat’s Pentachrome method. Lesions from BTK Inh. treated mice had higher collagen content (yellow-gold) and more uniform collagen density than control mice lesions. Lesions in MECA:siMMP-9 treated mice also showed greater collagen density relative to controls, but to a lesser extent than BTK Inh. treated animals. Intra-lesion ground/matrix substance positive staining (blue-green) was low in the innominate artery with no substantial differences observed between the groups (Fig 6A and S5 Fig). Representative Picro-Sirius red stained sections of aortic arch viewed under polarized light show collagen in atherosclerotic lesions found at the innominate artery of control and treated mice. Relative to control mice, lesions from MECA:siMMP-9 treated mice are smaller than other groups; lesions of both MECA:siMMP-9 and BTK Inh. treated mice had higher collagen content with more crosslinking/density (Fig 6B and S5 Fig).

**Fig 7:**
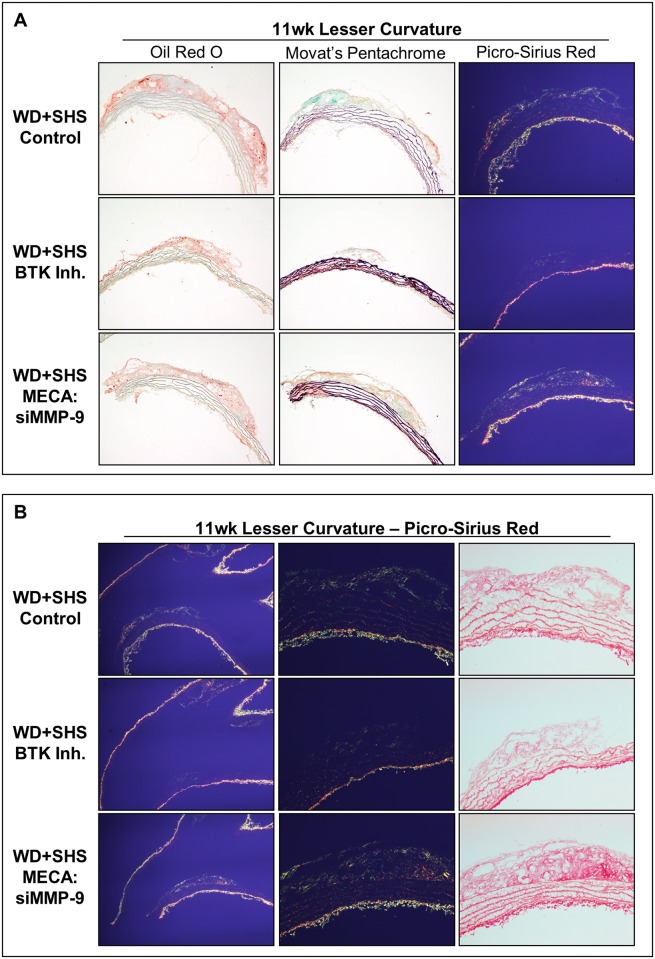
Histological analysis of aortic arch cross sections at the lesser curvature from ApoE^-/-^ mice following four week treatments. (A) Representative Oil Red O stained sections of aortic arch show atherosclerotic lesions found at the lesser curvature of the aortic arch in control and treated mice; 5–8 animals were analyzed per group. Neutral lipids stained red. Movat’s Pentachrome and Picro-Sirius red stained sections are also shown. Four control animals, 5 BTK inhibitor, and 5 MECA:siMMP-9 treated animals were evaluated. (B) Representative Picro-Sirius red stained sections of aortic arches show atherosclerotic lesions found at the lesser curvature of control and treated mice viewed under polarized light. Four control animals, 5 BTK inhibitor, and 5 MECA:siMMP-9 treated animals were evaluated.

Representative aortic arch sections stained with Oil Red O, Movat’s Pentachrome, and Picro-Sirius red are shown in Fig 7A and 7B. Analysis of Oil Red O stained lesions at the lesser curvature of control and treated mice showed that lesion size was reduced in BTK Inh. treated animals relative to control mice, and to a lesser extent in MECA:siMMP-9 treated animals (Fig 7A and S6 Fig). Picro-Sirius red staining and polarized light were also employed for analysis of the lesser curvature of the aortic arch from control and BTK Inh. treated mice. Lesions again appeared smaller relative to control mice lesions, but lesser curvature lesion collagen density was similar for all groups, with generally higher density relative to innominate artery lesions, but with intermittent regions of low collagen content at the lesion periphery (Fig 7B).

**Fig 8:**
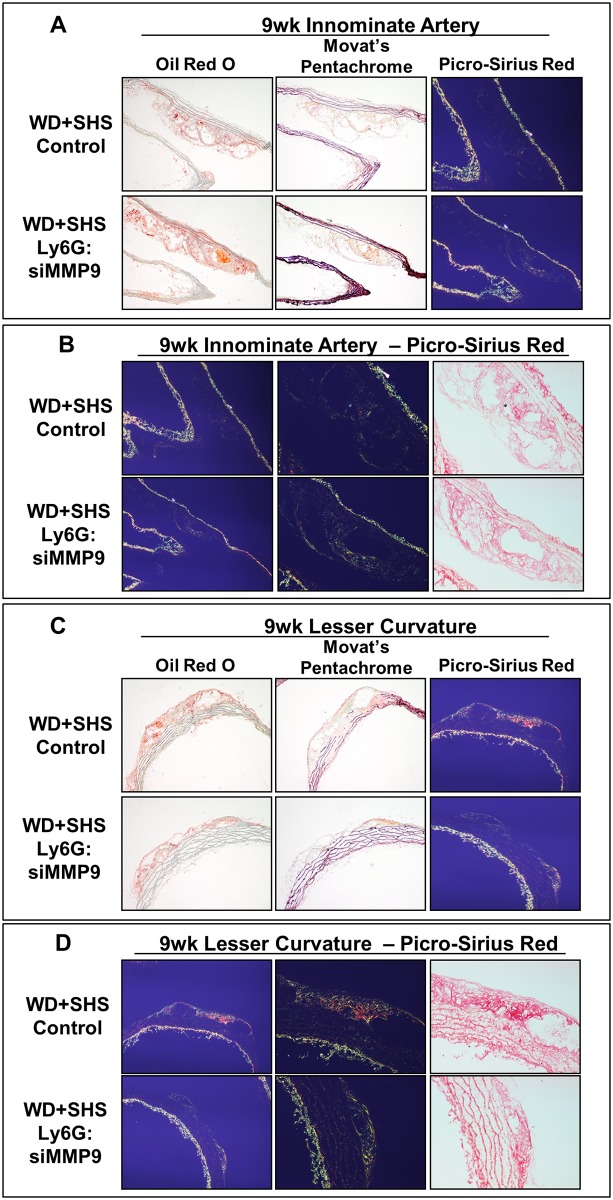
Histological analysis of aortic arch cross sections from ApoE^-/-^ mice following two week treatments. (A) Representative sections of aortic arch show atherosclerotic lesions found at the initial branch of the innominate artery of control and treated mice, 5 mice per group were analyzed. Movat’s Pentachrome and Picro-Sirius red stained sections are also shown. (B) Representative Picro-Sirius red stained sections of aortic arches show atherosclerotic lesions found at the innominate artery branch of control and treated mice when viewed under polarized light. (C) Representative sections of aortic arches show atherosclerotic lesions found at the lesser curvature of control and treated mice. Movat’s Pentachrome and Picro-Sirius red stained sections are also shown; 4–5 animals per group were evaluated. (D) Representative Picro-Sirius red stained sections of aortic arches show atherosclerotic lesions found at the lesser curvature in control and treated mice when viewed under normal (right) and polarized (left, center) light; 4–5 animals per group were evaluated.

Atherosclerotic lesions found at the innominate artery branch of control and treated mice were stained with Oil Red O, Movat’s Pentachrome, and PS red and are shown in Fig 8A and S7 Fig. Relative to control mice, lesion size was reduced in Ly6G:siMMP-9 treated animals and lesion collagen density appeared to improve in Movat stained sections. Intra-lesion ground/matrix substance positive staining (blue-green) was similar for both groups and generally low. Representative Picro-Sirius red stained sections of aortic arch show atherosclerotic lesions found at the innominate artery branch of control and treated mice when viewed under polarized light. Innominate artery branch lesion collagen density was similar for all groups, with generally higher density relative to lesser curvature lesions, but with intermittent regions of low collagen content, usually at the lesion periphery (Fig 8B).

Fig 8C and 8D show representative atherosclerotic lesions found at the lesser curvature of the aortic arch of control and treated mice stained with Oil Red O, Movat’s Pentachrome, and PS Red. Lesion size was reduced somewhat in treated animals relative to controls. Lesion collagen density varied within groups and was generally higher in treated mice than controls as indicated from Movat’s Pentachrome staining; collagen stained yellow-gold. Intra-lesion ground/matrix substance staining (blue-green) was similar for both groups and generally low (Fig 8C and S8 Fig).

**Fig 9:**
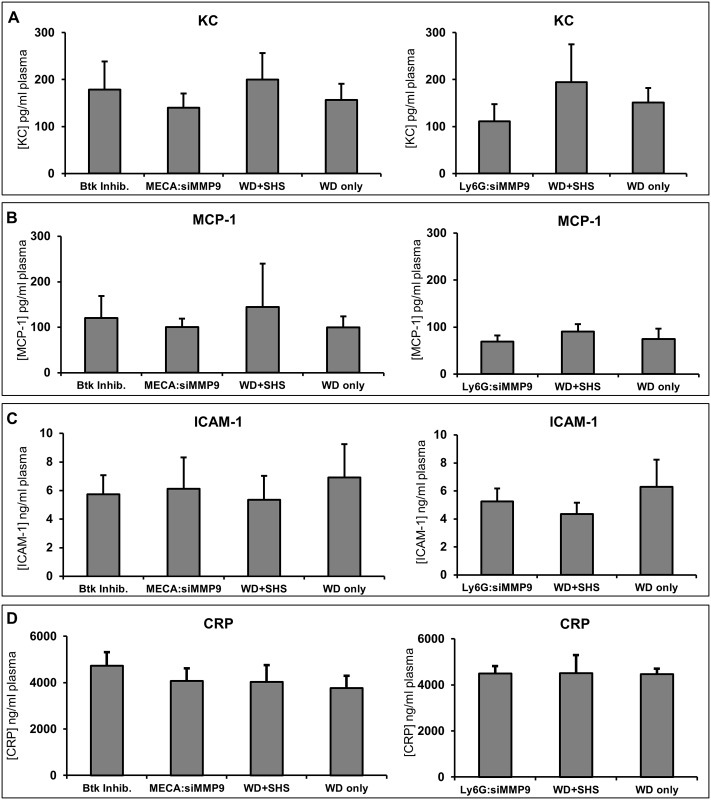
Changes in plasma levels of chemokines (KC, MCP-1), CRP, and ICAM-1. Changes in plasma levels of chemokines (KC and monocyte chemotactic protein-1 [MCP-1]), C-reactive protein (CRP), and adhesion molecules (intercellular adhesion molecule, ICAM-1) at 9 and 11 weeks post WD and / or SHS exposure with and without treatments. BTK Inh. n = 6, MECA n = 4, 11wk WD+SHS n = 9, 11wk WD only n = 5, 9wk WD+SHS n = 5, 9wk WD only n = 5, Ly6G n = 4.

We also evaluated the changes in plasma levels of chemokines (KC and MCP-1) (Fig 9A and 9B) as well as other inflammatory mediators, including CRP and ICAM-1 (Fig 9C and 9D). We did not observe significant differences between treated and untreated animals at 9 and 11 weeks post WD and / or SHS exposure.

**Fig 10:**
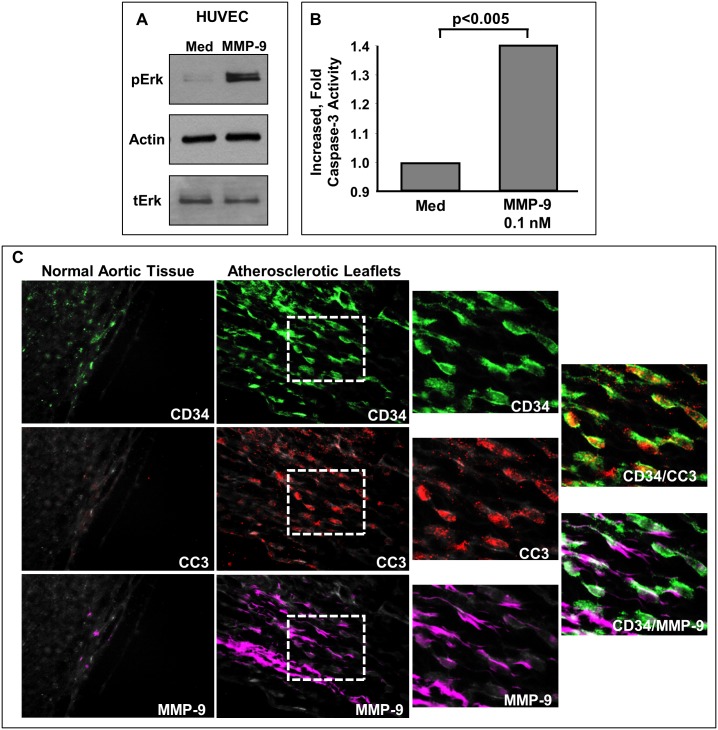
MMP-9 activation of endothelial cells. (A) Effect of stimulation with MMP-9 on HUVEC cell activation (pERK); n = 4. (B) Effect of stimulation with MMP-9 on HUVEC cell apoptosis (caspase-3 activity; p<0.005); n = 6 (C) Expression of MMP-9 and CC3 in aortic leaflets from patients with atherosclerosis; n = 3.

**Fig 11:**
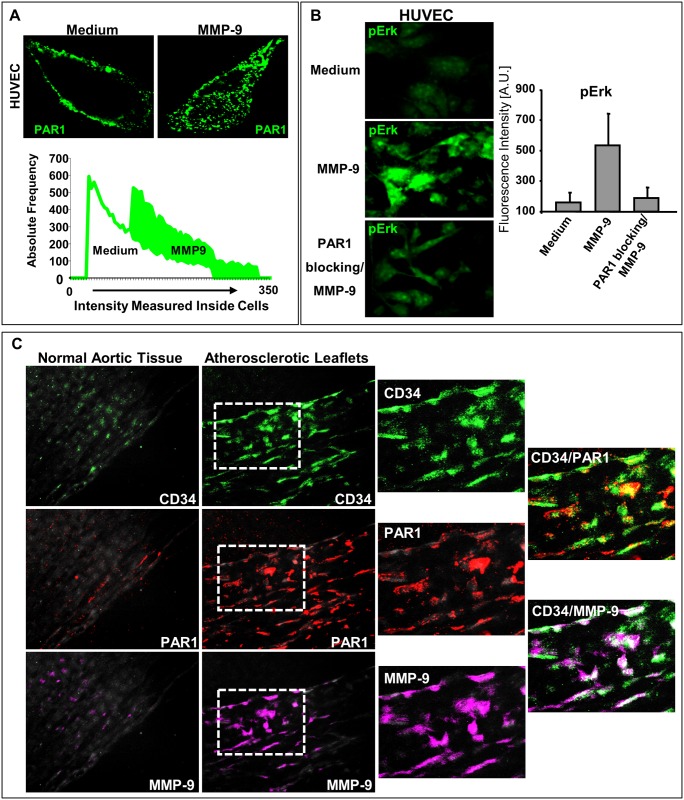
MMP-9 interaction with endothelial cell PAR-1. (A) Activation of PAR-1 by MMP-9 (pseudo color green); n = 3. (B) Expression of pERK in HUVEC cells by MMP-9 in presence and absence of anti-PAR-1; n = 3. (C). Expression of MMP-9 and PAR-1 in aortic leaflets from patients with atherosclerosis; n = 3.

Based on our findings, we hypothesized that MMP-9 directly affects endothelial cells. Therefore, we sought to determine the ability of MMP-9 to induce endothelial cell activation, and specifically increase in ERK activation. The ERK cascade regulates essential functions of endothelial cells, including apoptosis [21]. HUVEC cells were stimulated with MMP-9 and phosphorylated ERK was analyzed by Western blot, or confocal microscopy. We detected increased levels of pERK in cells treated with MMP-9 (Figs 10A and 11B). As apoptosis of endothelial cells contributes to endothelial injury and thrombosis in advanced atherosclerotic plaques [22], we also analyzed activation of caspase-3. The amount of cleaved (active) caspase-3 was increased in HUVEC cells stimulated with MMP-9 (Fig 10B). We also evaluated aortic leaflets obtained from atherosclerosis patients. We detected expression of enzymatically active MMP-9 (magenta) associated with endothelial cells (red) in these specimens (Fig 10C, bottom right panels), but not in normal tissue (Fig 10C, bottom left panel). Endothelial cells (CD34; green) were observed to be undergoing apoptosis, detected as positive staining for cleaved (active) caspase-3, CC3; red. The amount of CC3 was higher in aortic leaflets from patients with atherosclerosis (Fig 10C).

Next, we examined how MMP-9 interacts with endothelial cells. There is no known receptor for MMP-9; however, the direct comparison of amino acid sequences recognized by MMP-9 (the consensus / substrate recognition motif) and the amino acid sequence of the cleavage site generated by enzymatic activation of protease activated receptor-1 (PAR-1) shows considerable similarities [23–25]. As such, we hypothesized that MMP-9 could cleave and activate PAR-1. A classical thrombin receptor, PAR-1, is abundantly expressed on a variety of cells, including vascular endothelial cells. Upon activation, PAR-1 initiates cellular signaling via heterotrimeric G proteins. The primary function of PAR-1 is maintaining of normal homeostasis, but overexpression of this receptor has been linked to the pathogenesis of atherosclerosis and other human diseases [26, 27]. We found that MMP-9 has the ability to activate / cleave PAR-1 (pseudo color green) (Fig 11A). Using confocal laser microscopy and antibodies recognizing cleaved and intact PAR-1 (ATAP-2), the frequency and intensity of fluorescent signals monitored inside cells indicate that PAR-1 was cleaved by MMP-9 and internalized as depicted in Fig 11A. Accordingly, there is a shift in the intensity of the fluorescence signal detected inside endothelial cells, implicating the difference in the intensities of signals that were measured for a control sample (medium) and for sample treated with MMP-9 (PAR was labeled “green”). To confirm that MMP-9 can indeed activate PAR-1, we have performed blocking experiments. Treatment of endothelial cells with specific anti-PAR-1 antibodies (a mixture of ATAP-2 and WEDE-15) before stimulation with MMP-9 resulted in suppression of MMP-9 induced effects in these cells. Fig 11B shows the outcome of treatment with anti-PAR-1 antibodies on activation of ERK (levels of pERK).

There was also increased expression of PAR-1 (red) in atherosclerotic leaflets (Fig 11C). CD34 (green) was used as a marker of human endothelial cells. In addition, the levels of active MMP-9 (magenta) in endothelial cells were higher (Fig 11C).

## Discussion

Atherosclerosis is an underlying cause of the majority of cardiovascular diseases, including coronary artery disease, heart failure, and stroke. Impaired endothelial cell function is a hallmark of atherosclerosis. This endothelial dysfunction is directly associated with the development of atherosclerosis, and is present at all stages of the disease. Alterations in endothelial function precede morphological atherosclerotic changes [4–6, 28–31].

The recruitment of leukocytes, including neutrophils and monocytes, to the arterial wall is crucial for atherogenesis [4–6, 28–31]. Leukocytes and their products, such as MMP-9, contribute to atherosclerosis pathophysiology; however, the underlying mechanisms are not completely understood [7–9, 32].

We have evaluated MMP-9 concentration in ApoE^-/-^ mice plasma at 7 and 13 weeks post WD or regular diet. Because the exposure to SHS is a known contributor of the development of atherosclerosis, we also exposed half of the animals to SHS. We found that plasma concentrations of MMP-9 were elevated in these mice. We also noted that the levels of the active form of MMP-9 decreased in plasma over time. Finally, zymographic analysis of plasma from WD fed, SHS exposed, ApoE^-/-^ mice revealed significant bands in the molecular weight range corresponding to MMP-9:NGAL (neutrophil gelatinase-associated lipocalin) complexes. In humans, NGAL may protect MMP-9 from degradation [16–18] whereas in mice its function is less clear [33, 34].

In addition, specific therapies directed towards limiting expression of MMP-9 were protective in ApoE^-/-^ mice fed WD and exposed to SHS. These findings implicate an important role for MMP-9 in atherosclerosis. Moreover, we have shown that MMP-9 has the ability to induce apoptosis of endothelial cells and trigger activation of specific signaling pathways in these cells *in vitro*. Significantly, our results also indicate that MMP-9 exerts pro-inflammatory and pro-apoptotic activities towards endothelial cells through PAR-1. The stark correlation of increased PAR-1 and activated MMP-9 in atherosclerotic leaflets of human patients likewise support these conclusions. It should be emphasized that our novel observations advocate the activation/cleavage of PAR-1 by MMP-9 in atherosclerosis.

Both MMP-9 and PAR-1 contribute to pathophysiology of atherosclerosis and their expression is enhanced in individuals affected by this disease [26]. Enzymatic activation of PAR-1 results in cleavage of the receptor molecule between Arg 41 and Ser 42 [23]. The amino acid sequence of the cleavage site is P R S F L. The positions of these amino acids are marked P3 P_2_ P_1_ P_1_' P_2_' [24]. In addition, a recent study shows that MMP-9 has a preference for Arg at P_2_ and Ser or Thr at P_2_', and the consensus MMP-9 recognition motif is P R S/T Hy S/T (it is P_3_ through P_2_'), where Hy is a hydrophobic amino acid that could very well be F [25]. Taking into consideration the similarity of these sequences, we hypothesized that MMP-9 could cleave and activate PAR-1. Indeed, our findings support this notion.

The results of our studies suggest that MMP-9’s contribution to development of atherosclerotic lesions may be a direct stimulation of endothelial cells, and that PAR-1 may serve as a receptor for MMP-9.

## Supporting information

S1 Fig Aortic lesion area in ApoE-/- mice after 7 weeks exposure. Lesion area was significantly larger in WD + SHS exposed mice (n = 5) relative to all other groups (n = 3) at both the innominate artery and lesser curvature of the aortic arch, and Chow + SHS mice had significantly larger plaque areas than chow only mice at the lesser curvature (p<0.05). (A) When analyzing data based on individual sections rather than an average value for each animal WD only (n = 14) area at the lesser curvature was significantly larger than the chow only (n = 12) group (p<0.05). (B) Similarly when comparing measurements from individual sections at the innominate artery there was a tendency for WD only (n = 14) lesions to be larger than chow only (n = 12) lesions but the difference did not reach statistical significance (p = 0.098).
(PPTX)

S2 Fig Aortic lesion scoring in Movat’s pentachrome stained ApoE-/- aortic arch sections after 7 weeks exposure. (A) Collagen density in innominate artery plaques tended to score lower in SHS exposed groups than non-smoking, with a substantial difference between WD (n = 3) and WD + SHS (n = 4) groups, (p = 0.119). When analyzing data based on individual sections rather than an average value for each animal WD only density (n = 20) scored significantly higher than WD + SHS (n = 16) and Chow + SHS (n = 15) groups (p<0.05). (B) For matrix positive stain scoring no strong trends were observed for animal groups. When comparing individual section scores Chow only sections (n = 18) scored significantly higher than Chow + SHS (n = 15), WD + SHS (n = 16) and WD only (n = 20) sections (p<0.05) and WD only scored higher than WD + SHS (n = 16) but did not reach significance (p = 0.076).
(PPTX)

S3 Fig Aortic lesion area in ApoE-/- mice after 13 weeks exposure. (A) Innominate artery lesion size was significantly larger in WD+SHS mice (n = 6) than WD only (n = 4) and Chow only (n = 5) mice, and Chow+SHS mice (n = 4) had significantly larger plaques than WD only and Chow only mice. When comparing individual section measurements lesion size was larger in WD only (n = 25) and Chow only (n = 18) mice (p<0.05). There was also a difference between WD + SHS sections (n = 26) and Chow + SHS sections (n = 19) but the difference did not reach significance (p = 0.055). (B) Lesion area was significantly larger in WD + SHS exposed mice (n = 6) relative to all other groups (WD only n = 5, Chow only n = 5, and Chow + SHS n = 4) at the lesser curvature of the aortic arch, and Chow + SHS mice had significantly larger plaque areas than Chow only mice. Additionally, lesser curvature lesions in WD only mice were significantly larger than Chow only mice (p<0.05). (C) Collagen content measured in PS red stained sections was lower in the Chow + SHS group (n = 3) than the WD only group (n = 3, p<0.05). A similar trend was observed when comparing individual section measurements for WD + SHS (n = 18) and WD only (n = 13) but the difference was not significant (p = 0.198). Lower than expected values for percent collagen in Chow only animal lesions are likely due to the relative size (see S3 Fig A) and developmental state of the plaques being evaluated. Slower lesion growth in this group confounded attempts to compare their lesion collagen composition to age matched pro-atherogenic exposure mice.
(PPTX)

S4 Fig Aortic lesion scoring in Movat’s pentachrome stained ApoE-/- aortic arch sections after 13 weeks exposure. No clear trends were observed for animal groups based on Movat’s pentachrome stain histology scoring at either the innominate artery or the lesser curvature of the aortic arch. (A) When comparing individual tissue section collagen density scores the Chow only sections (n = 19) scored significantly higher than the Chow + SHS (n = 29) and WD + SHS (n = 31 sections), and WD only sections (n = 22) scored significantly higher than WD + SHS and Chow + SHS (p<0.05). WD + SHS scored substantially lower than Chow + SHS as well (p = 0.155). (B) When comparing individual tissue section matrix positive staining scores WD + SHS sections scored significantly lower than all other groups (p<0.05), and Chow + SHS scored lower than Chow only but the difference did not reach significance (p = 0.087).
(PPTX)

S5 Fig Innominate artery branch lesion scoring in ApoE-/- mouse aortic arch sections after 4 weeks of treatments. (A) Lesion areas measured in ORO stained sections were significantly higher in the control group (n = 8) than the BTK Inhib. (n = 5) and MECA:siMMP-9 (n = 4) groups (p<0.001). (B) Collagen content measured in PS red stained sections was lower in the control group (n = 3) than the MECA:siMMP-9 group (n = 4) but the difference did not reach significance (p = 0.062). When comparing individual section scores control sections (n = 14) collagen content was significantly lower than sections from BTK Inhib. (n = 18) and MECA:siMMP-9 (n = 22) treatment mouse sections (p<0.05). (C) Histology scoring from Movat stained sections indicated collagen density was lower in the control group (n = 3) than the BTK Inhib. treated (n = 5) group (p<0.05). When comparing individual sections the control group (n = 15) scored significantly lower than the two treatment groups (n = 19 each, p<0.05). (D) Matrix positive scoring from Movat stained sections did not reveal any trends between animal groups. When comparing individual section scores the control group (n = 15) scored lower than the MECA:siMMP-9 treatment group sections (n = 19) but the difference did not reach significance (p = 0.094).
(PPTX)

S6 Fig Lesser curvature lesion scoring in ApoE-/- mouse aortic arch sections after 4 weeks of treatments. (A) Lesion area measured in ORO stained sections of the aortic arch lesser curvature were similar for all groups of mice, control n = 8, BTK Inhib. and MECA:siMMP-9 treatment groups n = 5. When comparing individual section values control sections (n = 54) were larger than sections from BTK Inhib. treated (n = 23) animals but did not reach significance (p = 0.115). (B, C) In Movat stained sections both collagen density and matrix positive staining scores were substantially lower in the control group (n = 4) than the MECA:siMMP-9 treatment group (n = 5) but not significant (p = 0.117 and p = 0.113 respectively). When comparing individual section scores control sections (n = 19) were significantly lower than both BTK Inhib. (n = 22) and MECA:siMMP-9 (n = 19) treatment sections for both collagen density and matrix positive staining scores (p<0.05).
(PPTX)

S7 Fig Innominate artery branch lesion scoring in ApoE-/- mouse aortic arch sections after 2 weeks of treatments. (A) Lesion areas measured in ORO stained sections of the innominate artery branch were substantially higher in the control group (n = 5) compared to the group treated with Ly6G:siMMP-9 (n = 5, p = 0.178). When comparing individual measurements from the two groups lesion areas in control sections (n = 22) were significantly higher than in the treatment group (n = 26, p<0.01). (B) Collagen density scores from Movat staining were substantially higher for the Ly6G:siMMP-9 treated group (n = 5) compared to the control group (n = 5, p = 0.191) and when comparing individual section scores the treatment group (n = 20) scored significantly higher than the control group (n = 23, p<0.01). (C) Matrix positive staining scores were relatively low and similar for both groups.
(PPTX)

S8 Fig Lesser curvature lesion scoring in ApoE-/- mouse aortic arch sections after 2 weeks of treatments. (A) Lesion areas measured in ORO stained sections were similar for both groups. When comparing individual measurements from the two groups lesion areas in control sections (n = 22) were substantially larger than sections from Ly6G:siMMP-9 treated mice (n = 26, p = 0.113). (B, C) In Movat stained sections group results were similar between control (n = 4) and treatment (n = 5) animal groups for both collagen density and matrix positive staining scores. When comparing individual section collagen density scores control sections (n = 22) scored substantially lower than in the treatment group (n = 26, p = 0.096).
(PPTX)
