# Supplementary material for: Metalloproteinase-9 contributes to endothelial dysfunction in atherosclerosis via protease activated receptor-1
Source: PLoS One. 2017 Feb 6;12(2):e0171427. doi: 10.1371/journal.pone.0171427 (PMC5293219; doi:10.1371/journal.pone.0171427)
Supplement: S1 Fig — Lesion area was significantly larger in WD + SHS exposed mice (n = 5) relative to all other groups (n = 3) at both the innominate artery and lesser curvature of the aortic arch, and Chow + SHS mice had significantly larger plaque areas than chow only mice at the lesser curvature (p<0.05). (A) When analyzing data based on individual sections rather than an average value for each animal WD only (n = 14) area at the lesser curvature was significantly larger than the chow only (n = 12) group (p<0.05). (B) Similarly when comparing measurements from individual sections at the innominate artery there was a tendency for WD only (n = 14) lesions to be larger than chow only (n = 12) lesions but the difference did not reach statistical significance (p = 0.098). (PPTX) [file pone.0171427.s001.pptx]

## Slide 1
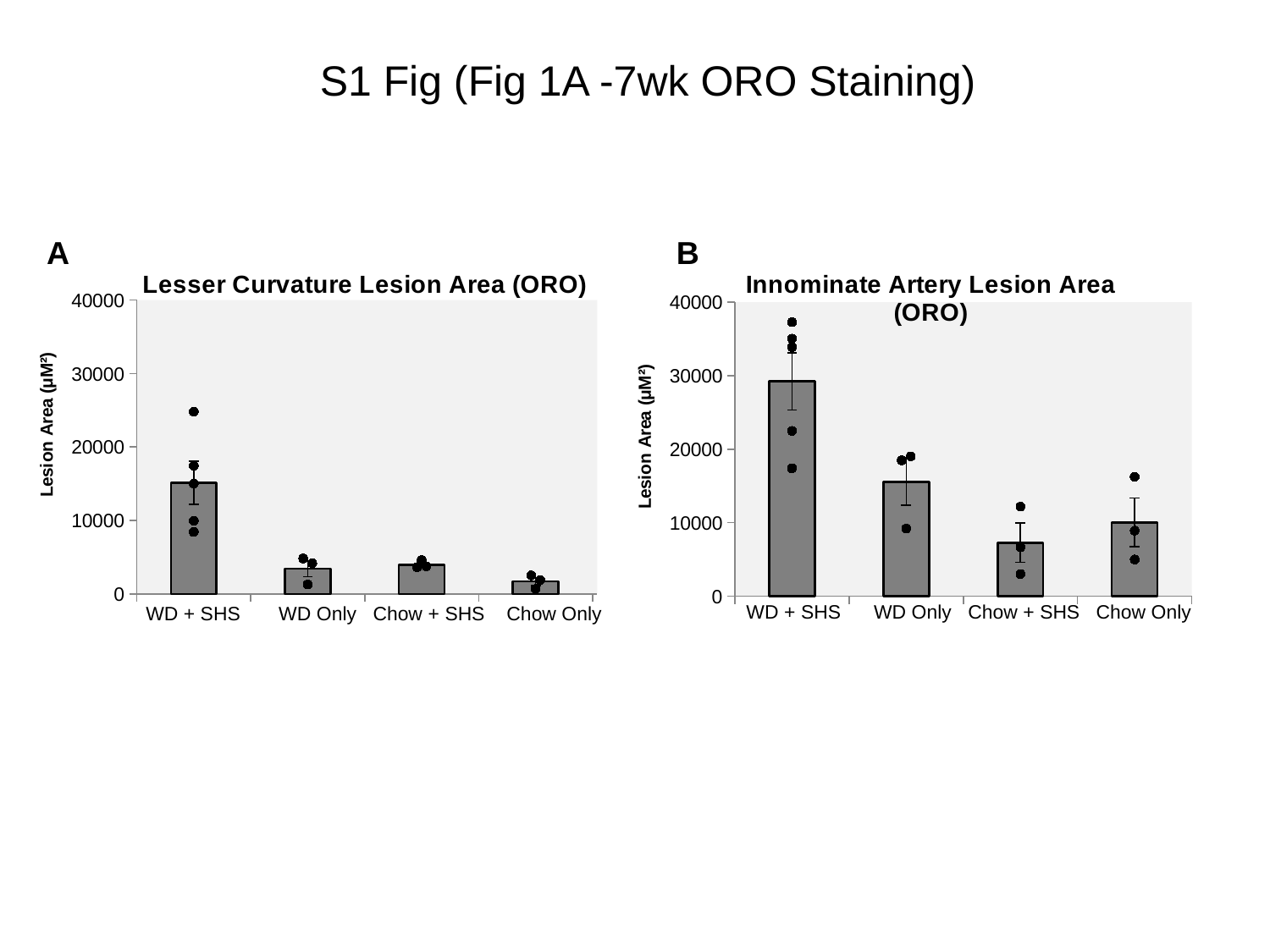

# S1 Fig (Fig 1A -7wk ORO Staining)
A
B
### Chart: Lesser Curvature Lesion Area (ORO)
| Category | | | | | | | |
|---|---|---|---|---|---|---|---|
| 1 | 15128.766666666666 | 9965.666666666666 | 8434.0 | 14995.666666666666 | 24801.25 | 17447.25 | None |
| 2 | 3444.972222222222 | 4833.25 | None | 4195.0 | 1306.6666666666667 | None | None |
| 3 | 4000.0833333333335 | 3641.25 | None | 4592.2 | 3766.8 | None | None |
| 4 | 1708.1666666666667 | None | 2528.5 | None | 1897.0 | 699.0 | None | WD + SHS WD Only Chow + SHS Chow Only
### Chart: Innominate Artery Lesion Area (ORO)
| Category | | | | | | | |
|---|---|---|---|---|---|---|---|
| 1 | 29225.5 | 35046.0 | 37297.0 | 33882.0 | 17402.75 | 22499.75 | None |
| 2 | 15570.516666666668 | 18474.75 | None | 9203.8 | 19033.0 | None | None |
| 3 | 7287.616666666666 | 6660.25 | None | 3018.0 | 12184.6 | None | None |
| 4 | 10056.25 | None | 8921.75 | None | 4999.0 | 16248.0 | None | WD + SHS WD Only Chow + SHS Chow Only
