# Supplementary material for: Metalloproteinase-9 contributes to endothelial dysfunction in atherosclerosis via protease activated receptor-1
Source: PLoS One. 2017 Feb 6;12(2):e0171427. doi: 10.1371/journal.pone.0171427 (PMC5293219; doi:10.1371/journal.pone.0171427)
Supplement: S2 Fig — (A) Collagen density in innominate artery plaques tended to score lower in SHS exposed groups than non-smoking, with a substantial difference between WD (n = 3) and WD + SHS (n = 4) groups, (p = 0.119). When analyzing data based on individual sections rather than an average value for each animal WD only density (n = 20) scored significantly higher than WD + SHS (n = 16) and Chow + SHS (n = 15) groups (p<0.05). (B) For matrix positive stain scoring no strong trends were observed for animal groups. When comparing individual section scores Chow only sections (n = 18) scored significantly higher than Chow + SHS (n = 15), WD + SHS (n = 16) and WD only (n = 20) sections (p<0.05) and WD only scored higher than WD + SHS (n = 16) but did not reach significance (p = 0.076). (PPTX) [file pone.0171427.s002.pptx]

## Slide 1
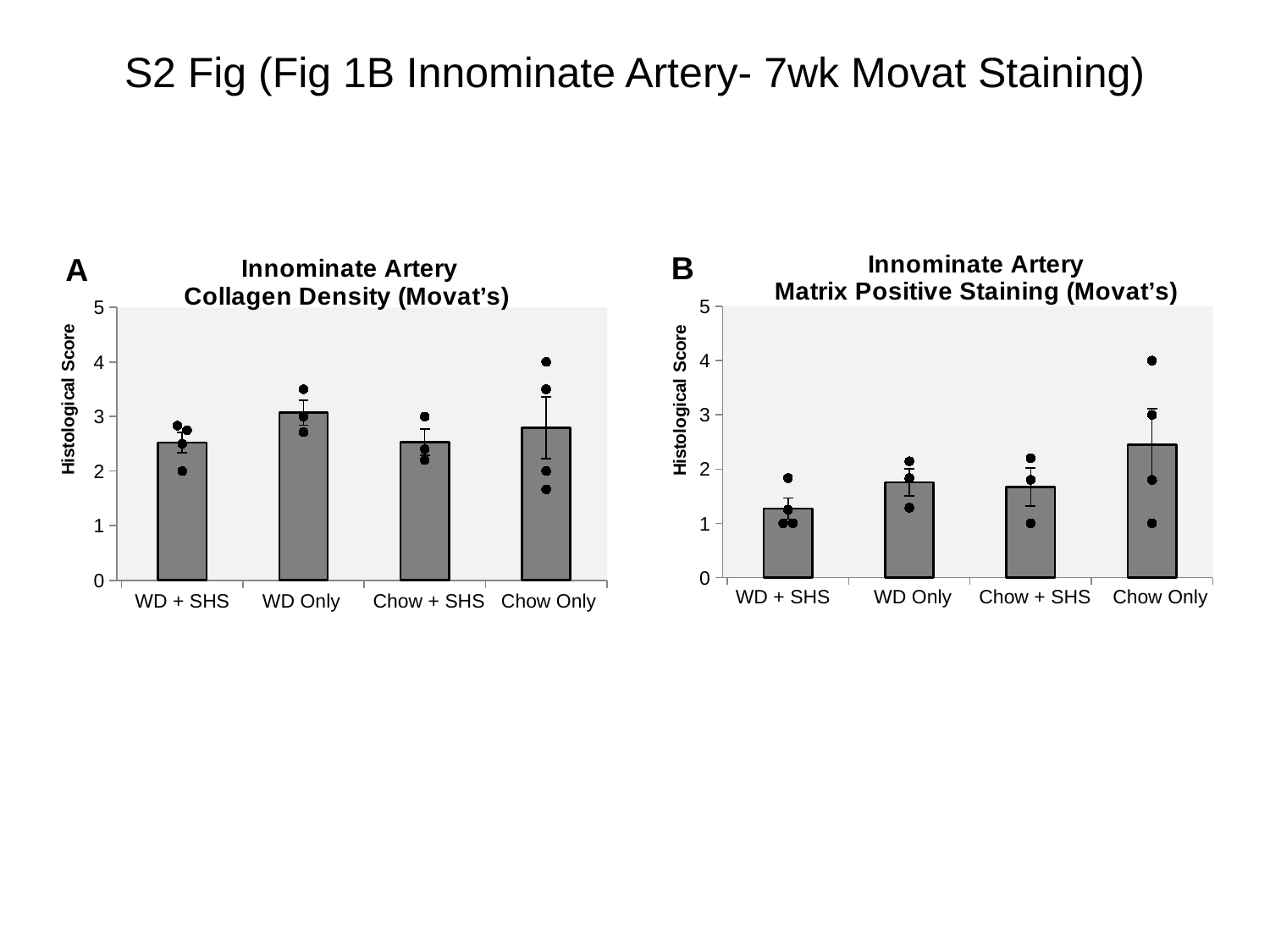

# S2 Fig (Fig 1B Innominate Artery- 7wk Movat Staining)
### Chart: Innominate Artery
Matrix Positive Staining (Movat’s)
| Category | | | | | | |
|---|---|---|---|---|---|---|
| 1 | 1.2708333333333333 | 1.0 | 1.25 | 1.8333333333333333 | 1.0 | None |
| 2 | 1.753968253968254 | 1.8333333333333333 | 1.2857142857142858 | 2.142857142857143 | None | None |
| 3 | 1.6666666666666667 | 1.0 | 2.2 | 1.8 | None | None |
| 4 | 2.45 | 1.0 | 4.0 | 1.8 | 3.0 | None | WD + SHS WD Only Chow + SHS Chow Only
B
A
### Chart: Innominate Artery
Collagen Density (Movat’s)
| Category | | | | | | |
|---|---|---|---|---|---|---|
| 1 | 2.5208333333333335 | 2.0 | 2.75 | 2.8333333333333335 | 2.5 | None |
| 2 | 3.0714285714285716 | 3.5 | 2.7142857142857144 | 3.0 | None | None |
| 3 | 2.533333333333333 | 2.2 | 3.0 | 2.4 | None | None |
| 4 | 2.791666666666667 | 1.6666666666666667 | 4.0 | 2.0 | 3.5 | None | WD + SHS WD Only Chow + SHS Chow Only
