# Supplementary material for: Metalloproteinase-9 contributes to endothelial dysfunction in atherosclerosis via protease activated receptor-1
Source: PLoS One. 2017 Feb 6;12(2):e0171427. doi: 10.1371/journal.pone.0171427 (PMC5293219; doi:10.1371/journal.pone.0171427)
Supplement: S3 Fig — (A) Innominate artery lesion size was significantly larger in WD+SHS mice (n = 6) than WD only (n = 4) and Chow only (n = 5) mice, and Chow+SHS mice (n = 4) had significantly larger plaques than WD only and Chow only mice. When comparing individual section measurements lesion size was larger in WD only (n = 25) and Chow only (n = 18) mice (p<0.05). There was also a difference between WD + SHS sections (n = 26) and Chow + SHS sections (n = 19) but the difference did not reach significance (p = 0.055). (B) Lesion area was significantly larger in WD + SHS exposed mice (n = 6) relative to all other groups (WD only n = 5, Chow only n = 5, and Chow + SHS n = 4) at the lesser curvature of the aortic arch, and Chow + SHS mice had significantly larger plaque areas than Chow only mice. Additionally, lesser curvature lesions in WD only mice were significantly larger than Chow only mice (p<0.05). (C) Collagen content measured in PS red stained sections was lower in the Chow + SHS group (n = 3) than the WD only group (n = 3, p<0.05). A similar trend was observed when comparing individual section measurements for WD + SHS (n = 18) and WD only (n = 13) but the difference was not significant (p = 0.198). Lower than expected values for percent collagen in Chow only animal lesions are likely due to the relative size (see S3 Fig A) and developmental state of the plaques being evaluated. Slower lesion growth in this group confounded attempts to compare their lesion collagen composition to age matched pro-atherogenic exposure mice. (PPTX) [file pone.0171427.s003.pptx]

## Slide 1
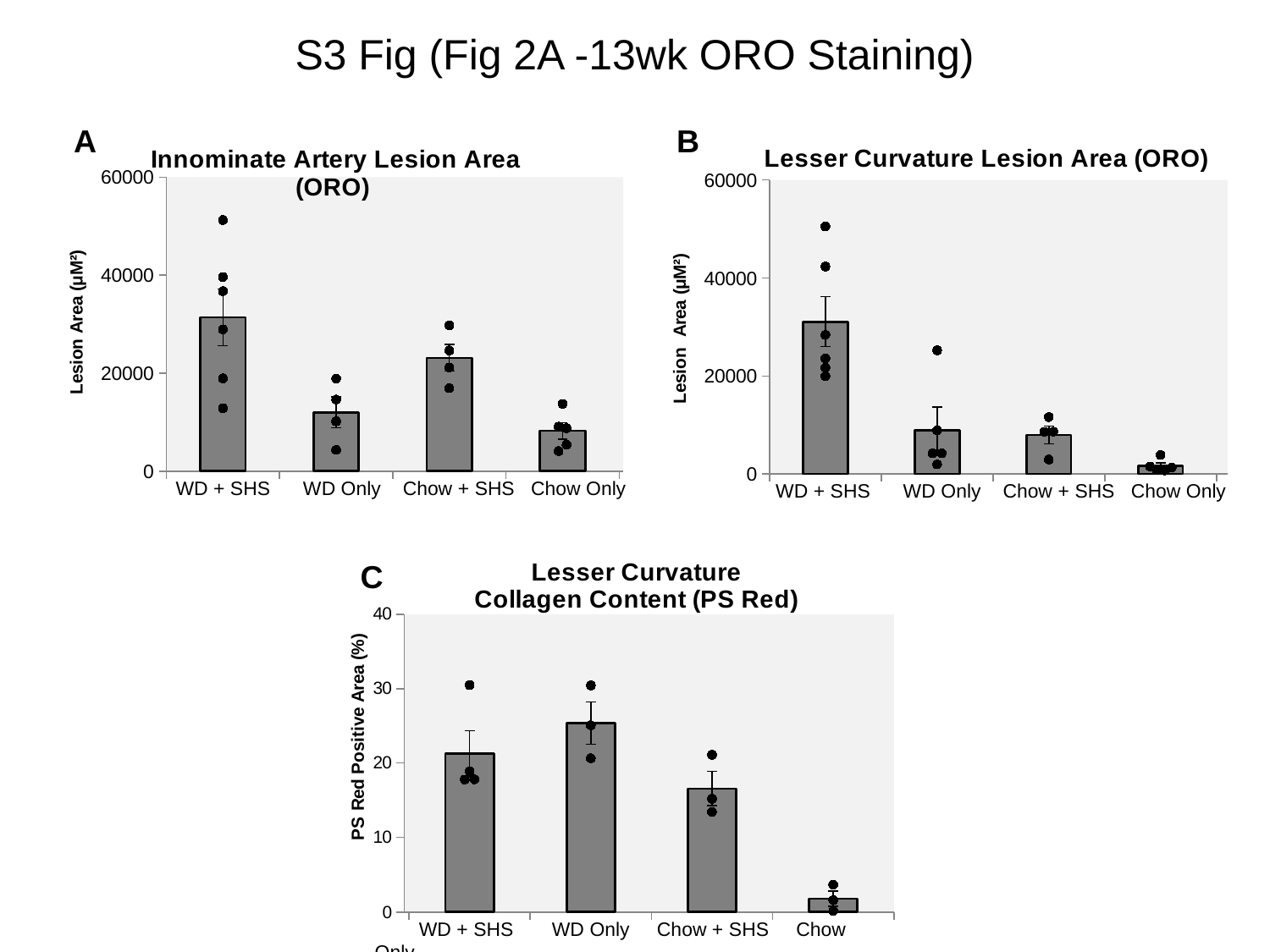

# S3 Fig (Fig 2A -13wk ORO Staining)
A
B
### Chart: Innominate Artery Lesion Area (ORO)
| Category | | | | | | | | |
|---|---|---|---|---|---|---|---|---|
| 1 | 31373.941666666666 | 12837.6 | 28903.0 | 18927.75 | 39613.8 | 36723.25 | 51238.25 | None |
| 2 | 11997.635416666666 | 4320.333333333333 | 18885.0 | 10178.333333333334 | 14606.875 | None | None | None |
| 3 | 23104.25 | 21135.6 | 24626.0 | 29750.0 | 16905.4 | None | None | None |
| 4 | 8209.816666666668 | 4094.0 | 9076.5 | 8762.5 | 13721.333333333334 | 5394.75 | None | None | WD + SHS WD Only Chow + SHS Chow Only
### Chart: Lesser Curvature Lesion Area (ORO)
| Category | | | | | | | | |
|---|---|---|---|---|---|---|---|---|
| 1 | 31062.191666666666 | 42332.0 | 21660.75 | 19940.5 | 23573.4 | 50496.5 | 28370.0 | None |
| 2 | 8907.755555555555 | 1952.6666666666667 | 4246.111111111111 | 8898.0 | 25232.0 | 4210.0 | None | None |
| 3 | 7945.820833333333 | 8611.4 | 11601.75 | 2949.8 | 8620.333333333334 | None | None | None |
| 4 | 1646.3166666666664 | 816.25 | 3874.5 | 1534.5 | 719.3333333333334 | 1287.0 | None | None | WD + SHS WD Only Chow + SHS Chow Only
### Chart: Lesser Curvature
Collagen Content (PS Red)
| Category | | | | | | |
|---|---|---|---|---|---|---|
| 1 | 21.24106932579027 | 18.896315585140485 | 17.782689673323272 | 17.807328512480552 | 30.477943532216766 | None |
| 2 | 25.38210423214444 | 25.07878247954208 | 20.638223335306183 | 30.429306881585045 | None | None |
| 3 | 16.57981477785881 | 15.189211854449146 | 21.114240776867188 | 13.435991702260106 | None | None |
| 4 | 1.7997968788599987 | 1.5919385423208832 | 0.1393360751654316 | 3.6681160190936812 | None | None | WD + SHS WD Only Chow + SHS Chow Only
C
