# Supplementary material for: Metalloproteinase-9 contributes to endothelial dysfunction in atherosclerosis via protease activated receptor-1
Source: PLoS One. 2017 Feb 6;12(2):e0171427. doi: 10.1371/journal.pone.0171427 (PMC5293219; doi:10.1371/journal.pone.0171427)
Supplement: S4 Fig — No clear trends were observed for animal groups based on Movat’s pentachrome stain histology scoring at either the innominate artery or the lesser curvature of the aortic arch. (A) When comparing individual tissue section collagen density scores the Chow only sections (n = 19) scored significantly higher than the Chow + SHS (n = 29) and WD + SHS (n = 31 sections), and WD only sections (n = 22) scored significantly higher than WD + SHS and Chow + SHS (p<0.05). WD + SHS scored substantially lower than Chow + SHS as well (p = 0.155). (B) When comparing individual tissue section matrix positive staining scores WD + SHS sections scored significantly lower than all other groups (p<0.05), and Chow + SHS scored lower than Chow only but the difference did not reach significance (p = 0.087). (PPTX) [file pone.0171427.s004.pptx]

## Slide 1
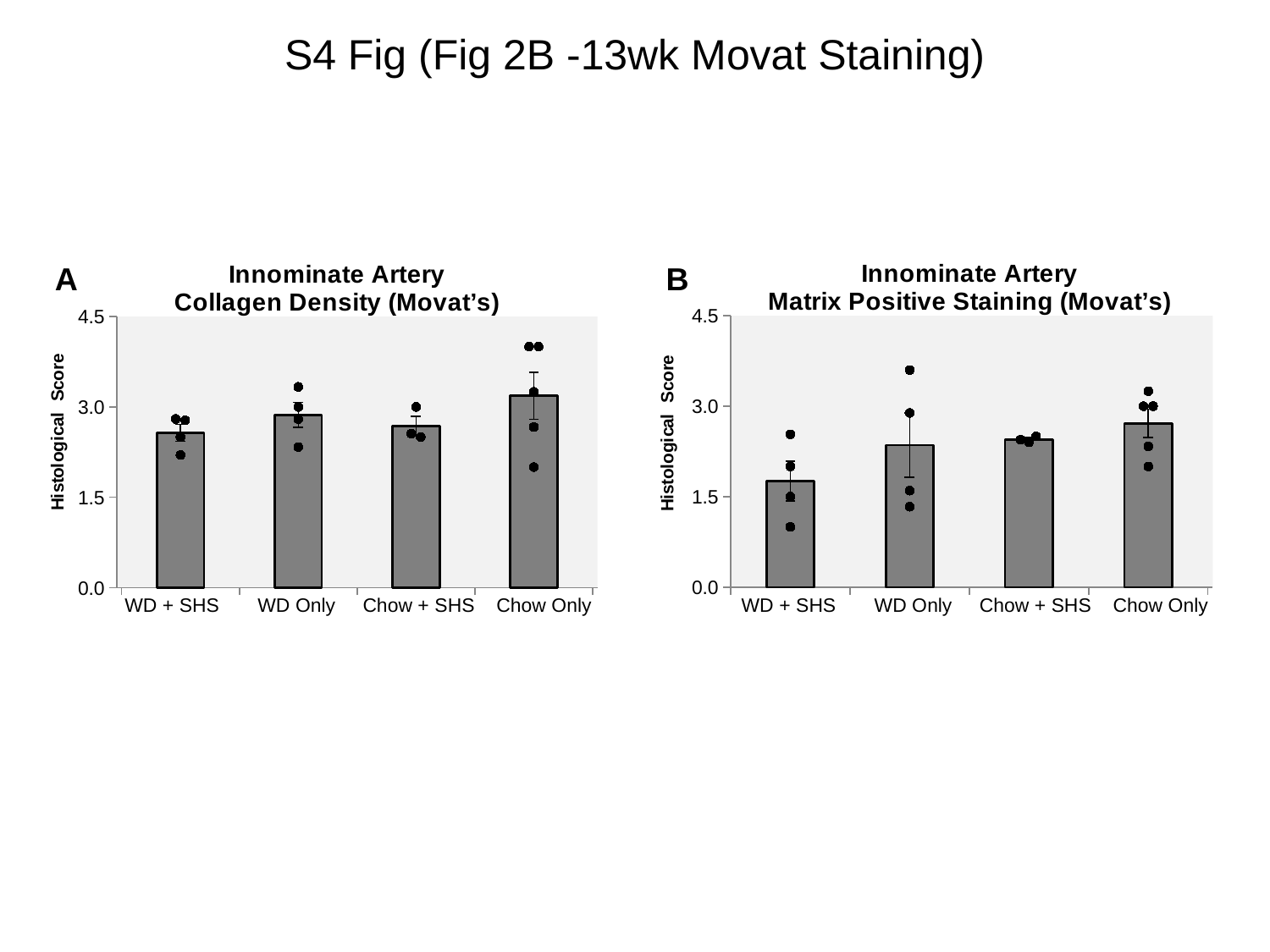

# S4 Fig (Fig 2B -13wk Movat Staining)
### Chart: Innominate Artery
Matrix Positive Staining (Movat’s)
| Category | | | | | | | |
|---|---|---|---|---|---|---|---|
| 1 | 1.7583333333333333 | 1.5 | 2.0 | 2.533333333333333 | 1.0 | None | None |
| 2 | 2.3555555555555556 | 1.3333333333333333 | 1.6 | 3.6 | 2.888888888888889 | None | None |
| 3 | 2.448148148148148 | 2.4 | 2.4444444444444446 | 2.5 | None | None | None |
| 4 | 2.716666666666667 | 2.3333333333333335 | 3.0 | 3.25 | 2.0 | 3.0 | None | WD + SHS WD Only Chow + SHS Chow Only
### Chart: Innominate Artery
Collagen Density (Movat’s)
| Category | | | | | | | |
|---|---|---|---|---|---|---|---|
| 1 | 2.5694444444444446 | 2.5 | 2.8 | 2.2 | 2.7777777777777777 | None | None |
| 2 | 2.8666666666666667 | 2.3333333333333335 | 3.0 | 2.8 | 3.3333333333333335 | None | None |
| 3 | 2.685185185185185 | 3.0 | 2.5555555555555554 | 2.5 | None | None | None |
| 4 | 3.183333333333333 | 2.6666666666666665 | 4.0 | 3.25 | 4.0 | 2.0 | None | WD + SHS WD Only Chow + SHS Chow Only
A
B
