# Supplementary material for: Metalloproteinase-9 contributes to endothelial dysfunction in atherosclerosis via protease activated receptor-1
Source: PLoS One. 2017 Feb 6;12(2):e0171427. doi: 10.1371/journal.pone.0171427 (PMC5293219; doi:10.1371/journal.pone.0171427)
Supplement: S5 Fig — (A) Lesion areas measured in ORO stained sections were significantly higher in the control group (n = 8) than the BTK Inhib. (n = 5) and MECA:siMMP-9 (n = 4) groups (p<0.001). (B) Collagen content measured in PS red stained sections was lower in the control group (n = 3) than the MECA:siMMP-9 group (n = 4) but the difference did not reach significance (p = 0.062). When comparing individual section scores control sections (n = 14) collagen content was significantly lower than sections from BTK Inhib. (n = 18) and MECA:siMMP-9 (n = 22) treatment mouse sections (p<0.05). (C) Histology scoring from Movat stained sections indicated collagen density was lower in the control group (n = 3) than the BTK Inhib. treated (n = 5) group (p<0.05). When comparing individual sections the control group (n = 15) scored significantly lower than the two treatment groups (n = 19 each, p<0.05). (D) Matrix positive scoring from Movat stained sections did not reveal any trends between animal groups. When comparing individual section scores the control group (n = 15) scored lower than the MECA:siMMP-9 treatment group sections (n = 19) but the difference did not reach significance (p = 0.094). (PPTX) [file pone.0171427.s005.pptx]

## Slide 1
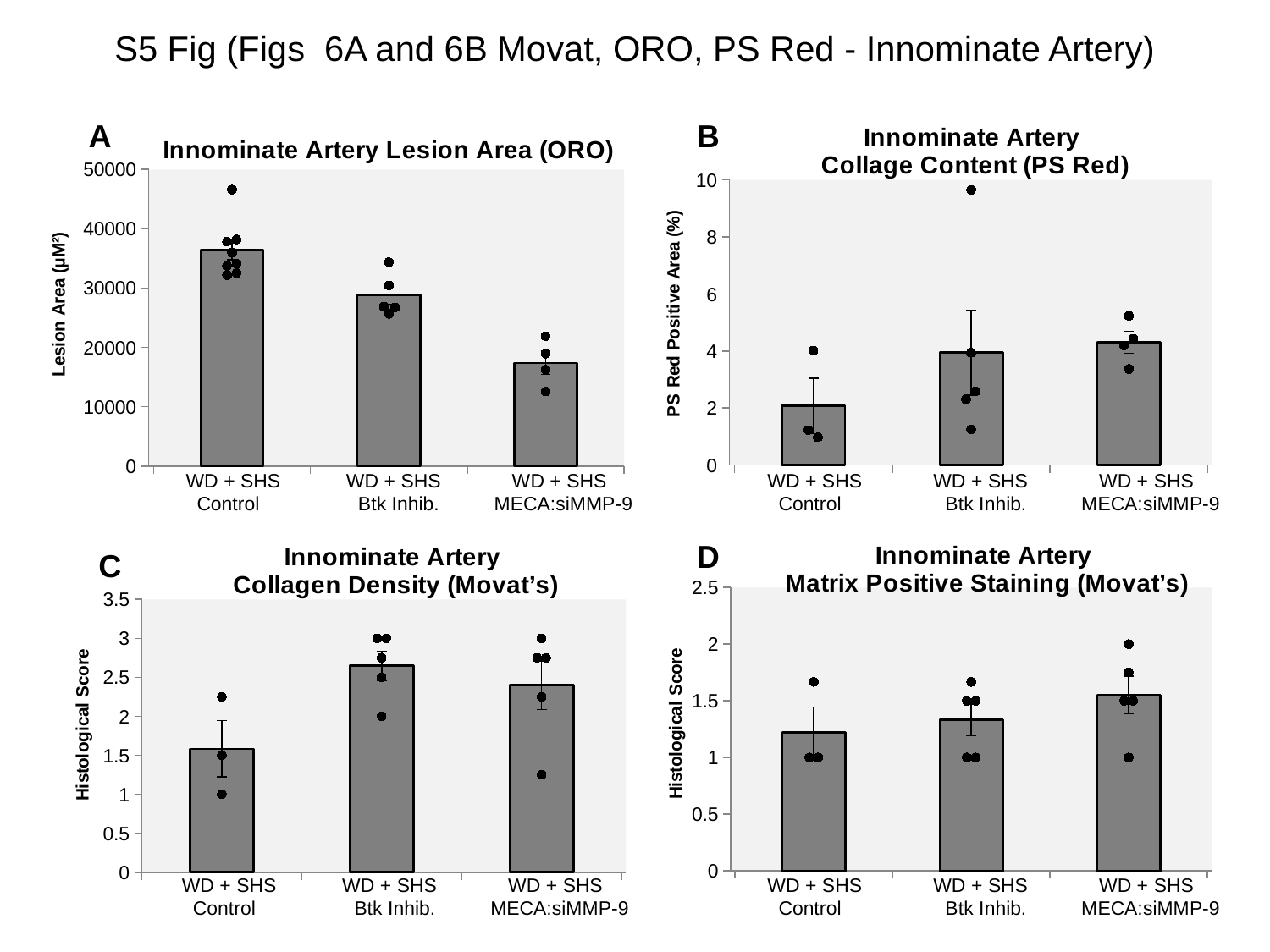

# S5 Fig (Figs 6A and 6B Movat, ORO, PS Red - Innominate Artery)
A
B
[unsupported chart]
### Chart: Innominate Artery Lesion Area (ORO)
| Category | | | | | | | | | | |
|---|---|---|---|---|---|---|---|---|---|---|
| 1 | 36386.946875 | 38184.4 | 32512.8 | 33757.25 | 37818.375 | 35959.375 | 32171.375 | 34102.5 | 46589.5 | None |
| 2 | 28805.93 | 25641.5 | 26863.75 | 34352.0 | 26723.4 | 30449.0 | None | None | None | None |
| 3 | 17387.425 | 21861.5 | 18926.0 | 16208.0 | 12554.2 | None | None | None | None | None | WD + SHS WD + SHS WD + SHS
 Control Btk Inhib. MECA:siMMP-9
 WD + SHS WD + SHS WD + SHS
 Control Btk Inhib. MECA:siMMP-9
D
### Chart: Innominate Artery
Matrix Positive Staining (Movat’s)
| Category | | | | | | | | |
|---|---|---|---|---|---|---|---|---|
| 1 | 1.2222222222222223 | 1.0 | 1.0 | 1.6666666666666667 | None | None | None | None |
| 2 | 1.3333333333333335 | 1.0 | 1.5 | 1.5 | 1.6666666666666667 | 1.0 | None | None |
| 3 | 1.55 | 1.75 | 1.0 | 1.5 | 1.5 | 2.0 | None | None |
### Chart: Innominate Artery
Collagen Density (Movat’s)
| Category | | | | | | | | |
|---|---|---|---|---|---|---|---|---|
| 1 | 1.5833333333333333 | 2.25 | 1.0 | 1.5 | None | None | None | None |
| 2 | 2.65 | 2.75 | 3.0 | 2.0 | 3.0 | 2.5 | None | None |
| 3 | 2.4 | 1.25 | 2.25 | 2.75 | 2.75 | 3.0 | None | None |C
 WD + SHS WD + SHS WD + SHS
 Control Btk Inhib. MECA:siMMP-9
 WD + SHS WD + SHS WD + SHS
 Control Btk Inhib. MECA:siMMP-9
