# Supplementary material for: Metalloproteinase-9 contributes to endothelial dysfunction in atherosclerosis via protease activated receptor-1
Source: PLoS One. 2017 Feb 6;12(2):e0171427. doi: 10.1371/journal.pone.0171427 (PMC5293219; doi:10.1371/journal.pone.0171427)
Supplement: S6 Fig — (A) Lesion area measured in ORO stained sections of the aortic arch lesser curvature were similar for all groups of mice, control n = 8, BTK Inhib. and MECA:siMMP-9 treatment groups n = 5. When comparing individual section values control sections (n = 54) were larger than sections from BTK Inhib. treated (n = 23) animals but did not reach significance (p = 0.115). (B, C) In Movat stained sections both collagen density and matrix positive staining scores were substantially lower in the control group (n = 4) than the MECA:siMMP-9 treatment group (n = 5) but not significant (p = 0.117 and p = 0.113 respectively). When comparing individual section scores control sections (n = 19) were significantly lower than both BTK Inhib. (n = 22) and MECA:siMMP-9 (n = 19) treatment sections for both collagen density and matrix positive staining scores (p<0.05). (PPTX) [file pone.0171427.s006.pptx]

## Slide 1
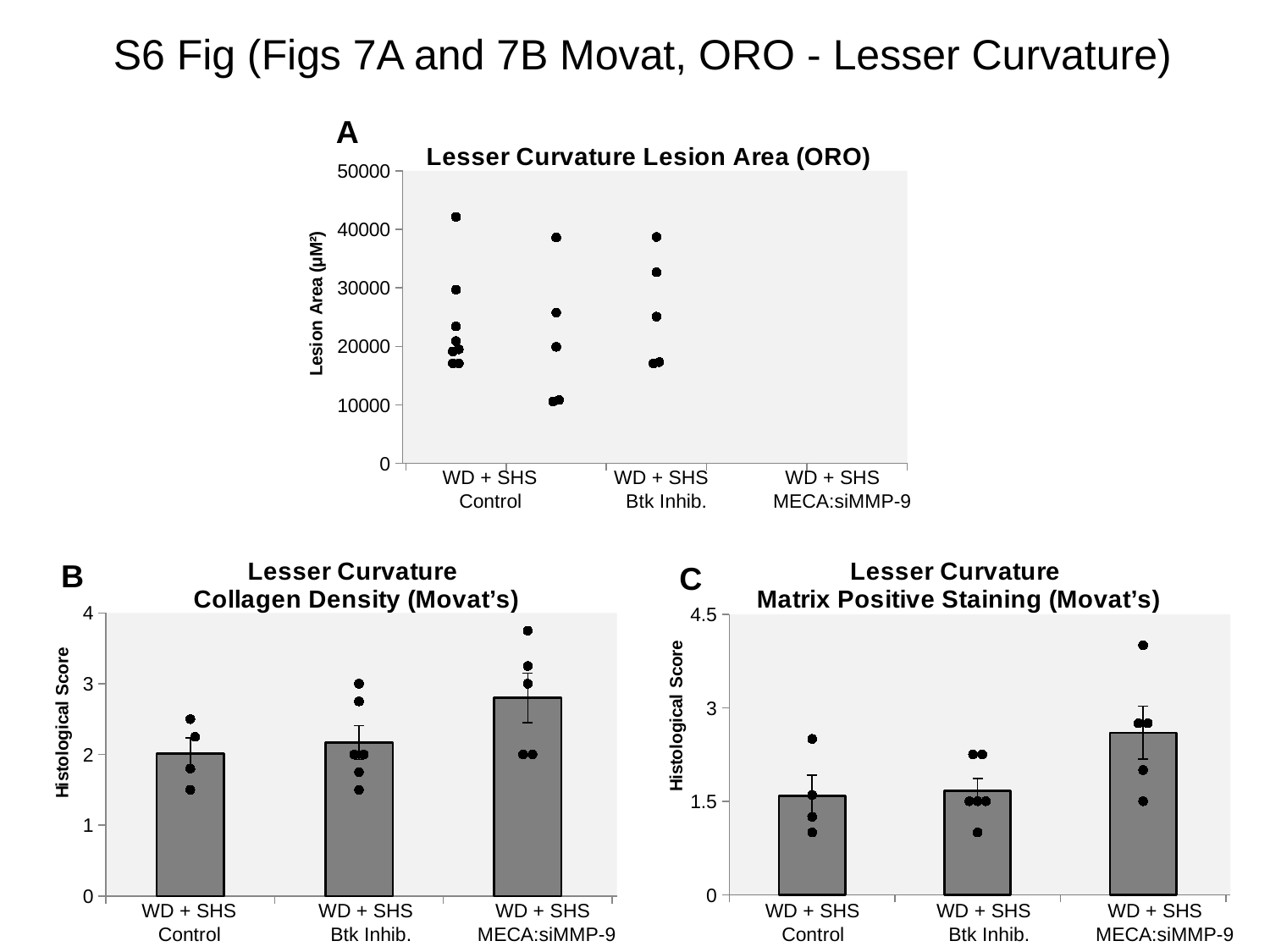

# S6 Fig (Figs 7A and 7B Movat, ORO - Lesser Curvature)
A
### Chart: Lesser Curvature Lesion Area (ORO)
| Category | | | | | | | | | | |
|---|---|---|---|---|---|---|---|---|---|---|
| 1 | 23614.934375 | 20901.6 | 19125.0 | 42126.75 | 23419.25 | 17094.25 | 17090.125 | 29676.25 | 19486.25 | None |
| 2 | 21134.760000000002 | 25776.25 | 38603.75 | 10815.2 | 19920.4 | 10558.2 | None | None | None | None |
| 3 | 26167.120000000003 | 32664.75 | 38700.0 | 17311.5 | 25086.6 | 17072.75 | None | None | None | None | WD + SHS WD + SHS WD + SHS
 Control Btk Inhib. MECA:siMMP-9
[unsupported chart]
 WD + SHS WD + SHS WD + SHS
 Control Btk Inhib. MECA:siMMP-9
B
[unsupported chart]
 WD + SHS WD + SHS WD + SHS
 Control Btk Inhib. MECA:siMMP-9
C
