# Supplementary material for: Metalloproteinase-9 contributes to endothelial dysfunction in atherosclerosis via protease activated receptor-1
Source: PLoS One. 2017 Feb 6;12(2):e0171427. doi: 10.1371/journal.pone.0171427 (PMC5293219; doi:10.1371/journal.pone.0171427)
Supplement: S7 Fig — (A) Lesion areas measured in ORO stained sections of the innominate artery branch were substantially higher in the control group (n = 5) compared to the group treated with Ly6G:siMMP-9 (n = 5, p = 0.178). When comparing individual measurements from the two groups lesion areas in control sections (n = 22) were significantly higher than in the treatment group (n = 26, p<0.01). (B) Collagen density scores from Movat staining were substantially higher for the Ly6G:siMMP-9 treated group (n = 5) compared to the control group (n = 5, p = 0.191) and when comparing individual section scores the treatment group (n = 20) scored significantly higher than the control group (n = 23, p<0.01). (C) Matrix positive staining scores were relatively low and similar for both groups. (PPTX) [file pone.0171427.s007.pptx]

## Slide 1
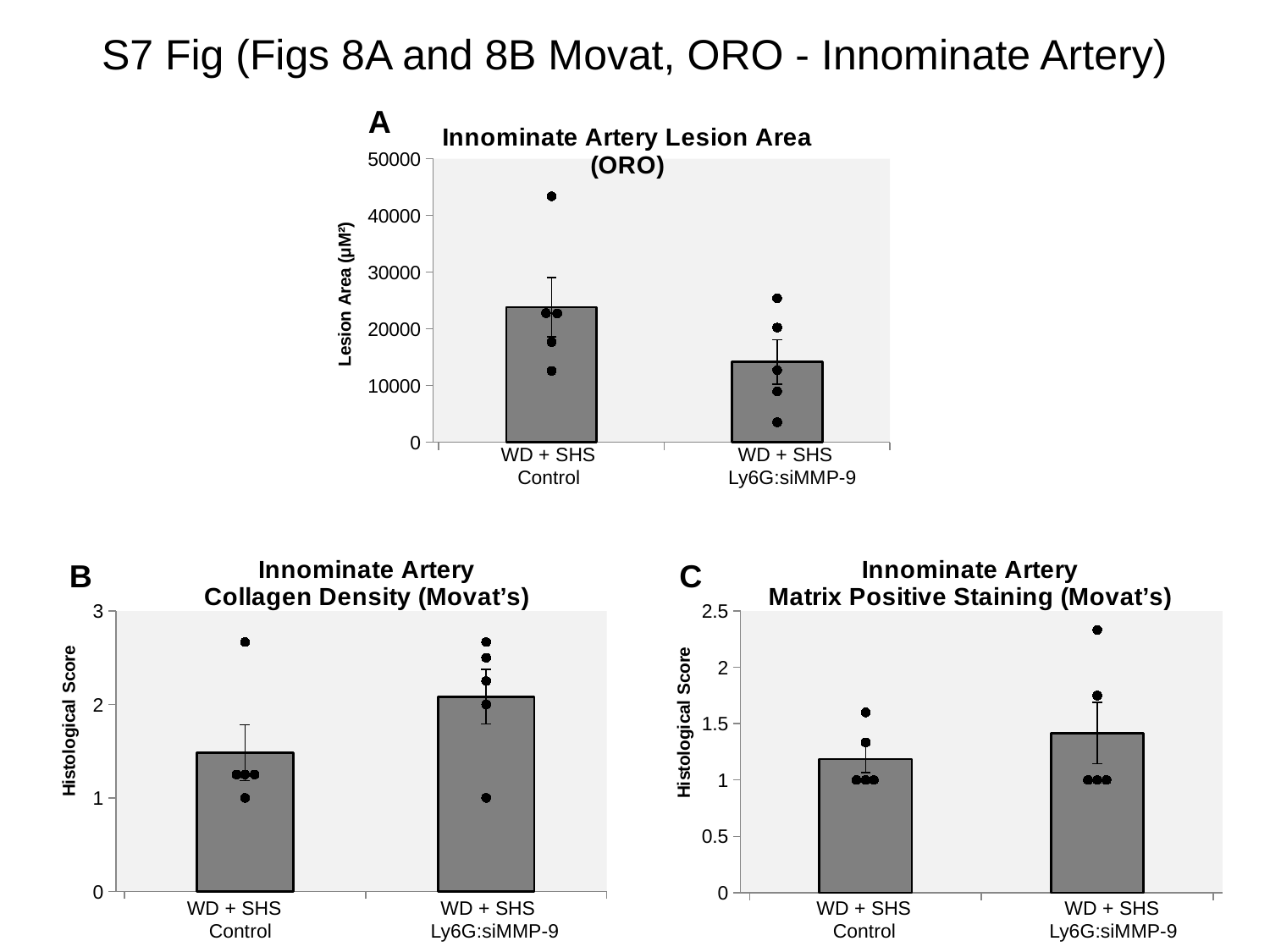

# S7 Fig (Figs 8A and 8B Movat, ORO - Innominate Artery)
A
### Chart: Innominate Artery Lesion Area (ORO)
| Category | | | | | | | |
|---|---|---|---|---|---|---|---|
| 1 | 23830.97 | 12583.0 | 22782.5 | 22737.75 | 43390.0 | 17661.6 | None |
| 2 | 14170.355 | 3539.75 | 25402.0 | 8963.8 | 20225.6 | 12720.625 | None | WD + SHS WD + SHS
 Control Ly6G:siMMP-9
### Chart: Innominate Artery
Matrix Positive Staining (Movat’s)
| Category | | | | | | | |
|---|---|---|---|---|---|---|---|
| 1 | 1.1866666666666668 | 1.0 | 1.0 | 1.0 | 1.3333333333333333 | 1.6 | None |
| 2 | 1.4166666666666667 | 1.0 | 1.0 | 1.0 | 2.3333333333333335 | 1.75 | None | WD + SHS WD + SHS
 Control Ly6G:siMMP-9
### Chart: Innominate Artery
Collagen Density (Movat’s)
| Category | | | | | | | |
|---|---|---|---|---|---|---|---|
| 1 | 1.4833333333333332 | 1.25 | 1.25 | 1.25 | 2.6666666666666665 | 1.0 | None |
| 2 | 2.083333333333333 | 2.25 | 2.5 | 2.0 | 2.6666666666666665 | 1.0 | None | WD + SHS WD + SHS
 Control Ly6G:siMMP-9
B
C
