# Supplementary material for: Metalloproteinase-9 contributes to endothelial dysfunction in atherosclerosis via protease activated receptor-1
Source: PLoS One. 2017 Feb 6;12(2):e0171427. doi: 10.1371/journal.pone.0171427 (PMC5293219; doi:10.1371/journal.pone.0171427)
Supplement: S8 Fig — (A) Lesion areas measured in ORO stained sections were similar for both groups. When comparing individual measurements from the two groups lesion areas in control sections (n = 22) were substantially larger than sections from Ly6G:siMMP-9 treated mice (n = 26, p = 0.113). (B, C) In Movat stained sections group results were similar between control (n = 4) and treatment (n = 5) animal groups for both collagen density and matrix positive staining scores. When comparing individual section collagen density scores control sections (n = 22) scored substantially lower than in the treatment group (n = 26, p = 0.096). (PPTX) [file pone.0171427.s008.pptx]

## Slide 1
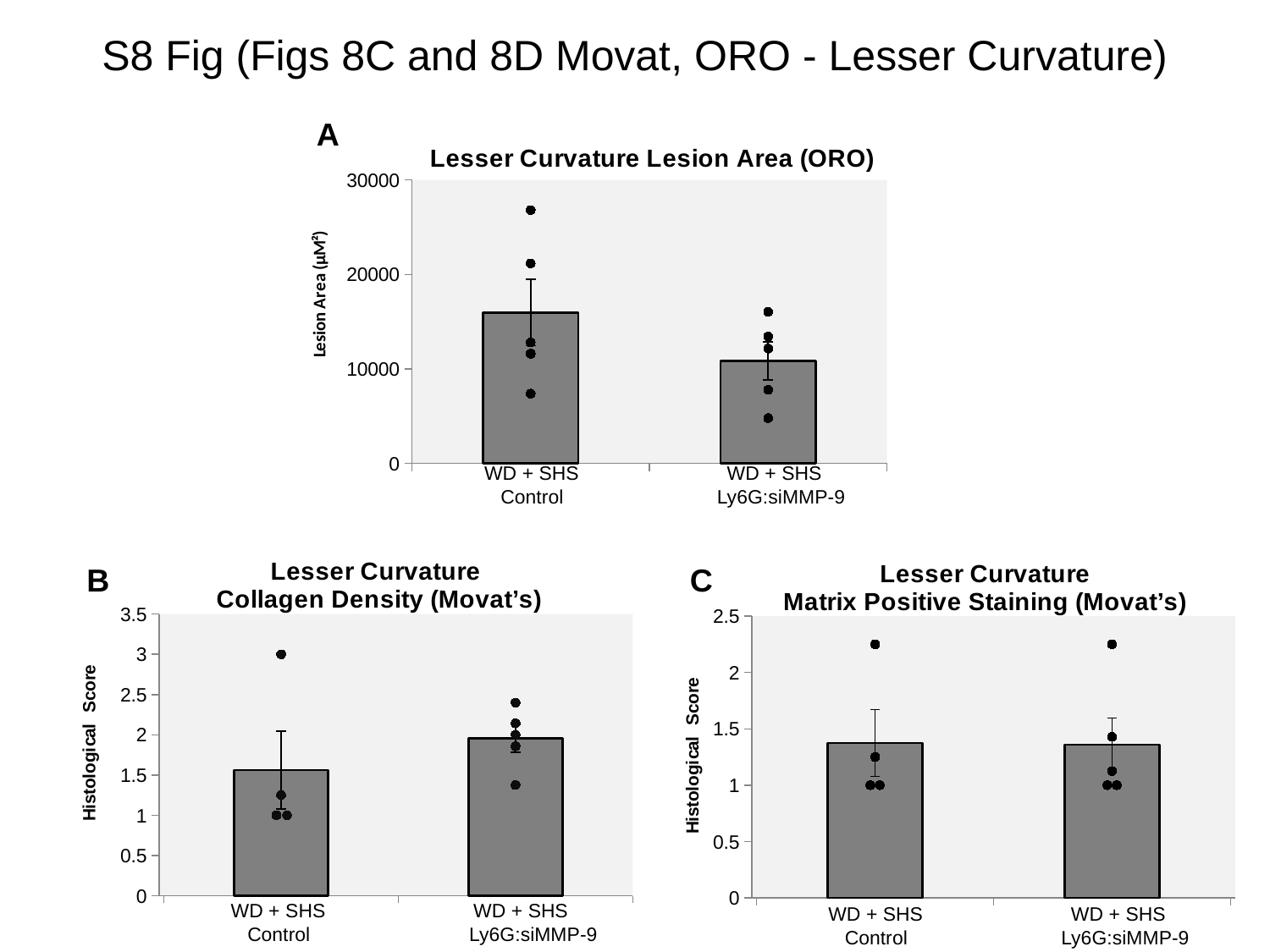

# S8 Fig (Figs 8C and 8D Movat, ORO - Lesser Curvature)
A
### Chart: Lesser Curvature Lesion Area (ORO)
| Category | | | | | | | |
|---|---|---|---|---|---|---|---|
| 1 | 15954.670000000002 | 21169.5 | 11610.25 | 12797.0 | 26812.8 | 7383.8 | None |
| 2 | 10839.369999999999 | 12151.0 | 7791.25 | 4794.8 | 13418.8 | 16041.0 | None | WD + SHS WD + SHS
 Control Ly6G:siMMP-9
### Chart: Lesser Curvature
Collagen Density (Movat’s)
| Category | | | | | | | |
|---|---|---|---|---|---|---|---|
| 1 | 1.5625 | 3.0 | 1.0 | 1.25 | 1.0 | None | None |
| 2 | 1.955 | 1.8571428571428572 | 2.0 | 2.4 | 2.142857142857143 | 1.375 | None | WD + SHS WD + SHS
 Control Ly6G:siMMP-9
### Chart: Lesser Curvature
Matrix Positive Staining (Movat’s)
| Category | | | | | | | |
|---|---|---|---|---|---|---|---|
| 1 | 1.375 | 2.25 | 1.25 | 1.0 | 1.0 | None | None |
| 2 | 1.3607142857142858 | 1.0 | 2.25 | 1.0 | 1.4285714285714286 | 1.125 | None | WD + SHS WD + SHS
 Control Ly6G:siMMP-9
B
C
